# Developmental Co-expression of Vglut2 and Nurr1 in a Mes-Di-Encephalic Continuum Preceeds Dopamine and Glutamate Neuron Specification

**DOI:** 10.3389/fcell.2019.00307

**Published:** 2019-11-28

**Authors:** Sylvie Dumas, Åsa Wallén-Mackenzie

**Affiliations:** ^1^Oramacell, Paris, France; ^2^Department of Organismal Biology, Unit of Comparative Physiology, Uppsala University, Uppsala, Sweden

**Keywords:** Vglut2, Nurr1, dopamine, glutamate, heterogeneity, subtype, Viaat, Vmat2

## Abstract

Midbrain dopamine (DA) neurons exist as several subtypes and are found in a heterogeneous environment including GABAergic and glutamatergic neurons as well as various types of co-releasing neurons. Developmental programs underlying this heterogeneity have remained elusive. In this study, combinatorial mRNA analysis was performed at stages when neuronal phenotypes are first specified. Vesicular transporters for dopamine and other monoamines (VMAT2), GABA (VIAAT), and glutamate (VGLUT2) were assessed by systematically applying fluorescent *in situ* hybridization through the mes-di-encephalon of the mouse embryo at embryonal days (E) 9.5–14.5. The results show that early differentiating dopamine neurons express the gene encoding VGLUT2 before onset of any dopaminergic markers. Prior to its down-regulation in maturing dopamine neurons, Vglut2 mRNA co-localizes extensively with Tyrosine hydroxylase (Th) and Nurr1, commonly used as markers for DA neurons. Further, Vglut2 and Nurr1 mRNAs are shown to overlap substantially in diencephalic neurons that maintain a glutamatergic phenotype. The results suggest that Vglut2/Nurr1-double positive cells give rise both to dopaminergic and glutamatergic neurons within the mes-di-encephalic area. Finally, analysis of markers representing subtypes of dopamine neurons, including the newly described NeuroD6 subtype, shows that certain subtype specifications arise early. Histological findings are outlined in the context of neuroanatomical concepts and the prosomeric model of brain development. The study contributes to the current decoding of the recently discovered heterogeneity among neurons residing along the cephalic flexure.

## Introduction

The midbrain dopamine (DA) system, originally described in the 1960’s, is a key brain substrate at the intersection between emotional, cognitive, and motor functions. In the mature rodent and primate, midbrain DA (mDA) neurons are located in the ventral aspect of the midbrain (also known as mesencephalon) where they are distributed in the ventral tegmental area (VTA) and substantia nigra *pars compacta* (SNc) (Dahlström and Fuxe, 1964). Crucial for reward and motivation, dysregulation of VTA DA neurocircuitry is implicated in neuropsychiatric conditions including schizophrenia and addiction, whereas degeneration of SNc DA neurons is a core feature of Parkinson’s disease (PD) (Björklund and Dunnett, 2007). Major focus has been aimed at decoding the developmental programs of mDA neurons, not least due to the strong interest in achieving rescue or *de novo* production of DA neurons for cell-replacement therapy in PD. Developmental programs required for specification, differentiation, migration and survival of mDA neurons depend on a tightly regulated cascade of soluble molecules and transcription factors (Hegarty et al., 2013; Arenas et al., 2015; Smidt, 2017). Following regional specification at the ventral midline of the mesencephalon, mDA progenitors have been described to start expressing transcription factor genes *Nurr1* and *Pitx3* which together with additional molecules activate the mDA phenotype. A critical step is the initiation of expression of the *Th* gene encoding Tyrosine hydoxylase, catalyzing the rate-limiting step in DA synthesis, and other genes defining a dopaminergic identity. Nurr1, Pitx3 and Th are found in all terminally differenting mDA neurons, however, this neuronal population is far from homogeneous. Afferent and efferent projections, electrophysiological patterns and responsiveness to sensory stimuli enable classification into distinct mDA subtypes (Morales and Margolis, 2017). Further, since VTA DA neurons have been found less susceptible than SNc DA neurons in PD, gene expression differences have been explored which has enabled identification of candidate genes that may confer neuroprotective properties (Chung et al., 2005; Greene et al., 2005; Viereckel et al., 2016). Recently, through technical advancements allowing for mRNA sequencing from single cells (scRNAseq), analysis of stem cell, murine and human DA neurons has led to the identification of molecularly defined subtypes of both developing and mature DA neurons (Poulin et al., 2014; La Manno et al., 2016; Kee et al., 2017; Tiklová et al., 2019). These findings will likely form the foundation for a new generation of knowledge of how to generate and to distinguish between mDA neurons.

Parallel to the increasing awareness of heterogeneity in the mDA population, it has become evident that the local environment of mDA neurons is strongly heterogeneous (Pupe and Wallén-Mackenzie, 2015; Morales and Margolis, 2017). GABAergic and glutamatergic neurons are intermingled with mDA neurons, and their role in the mature brain is currently under intense investigation: By either locally affecting mDA neurons and/or by participating in similar circuitries, their function is crucial for dopaminergic activities, and behavioral regulation (Hnasko et al., 2012; Tan et al., 2012; Wang et al., 2015; Yoo et al., 2016; Root et al., 2018, 2014). Further increasing the level of heterogeneity, some mDA neurons do themselves co-release GABA or glutamate. The mechanism for GABA co-release remains to be fully clarified but appears to involve the vesicular monoamine transporter (VMAT2) (Tritsch et al., 2016), while expression of the gene encoding the vesicular glutamate transporter 2 (VGLUT2) enables identification of glutamate-signaling neurons (Yamaguchi et al., 2011). Expression of the *Vglut2* gene within mDA neurons varies with age, and gene-knockout of *Vglut2* in DA neurons significantly alters dopaminergic function (Birgner et al., 2010; Hnasko et al., 2010; Stuber et al., 2010; Alsiö et al., 2011; Fortin et al., 2012; Wang et al., 2017; Papathanou et al., 2018). Recently, a newly identified subtype of mDA neurons defined by expression of *NeuroD6* (Viereckel et al., 2016; Khan et al., 2017; Kramer et al., 2018) was shown to partially co-express *Th* with *Vglut2*, and to play a distinct role in behavioral reinforcement (Bimpisidis et al., 2019). Despite the strong functional implication of the role of VGLUT2-positive mesencephalic neurons, their developmental process has remained largely unexplored.

The developmental heterogeneity of the brain habitat surrounding mDA neurons is crucial to decode, not least as it could aid in improving differentiation and survival protocols aimed toward improved treatment prospects of disorders that implicate these neurons. Here, we implemented systematic histological analysis of neurons residing along the cephalic flexure to address heterogeneity around the developmental stages when mDA neurons are first defined and subtypes formed. Our findings point toward an additional level of unexpected heterogeneity as most ventral cells of both the mesencephalon and diencephalon are shown to co-express the glutamatergic marker *Vglut2* and the dopaminergic marker *Nurr1* in early development. Vglut2 and Nurr1 mRNAs, along with NeuroD6 and additional markers of mDA subtypes, are addressed in the context of gene expression patterns relevant for the local habitat of mes-and-diencephalic DA and glutamate neurons. Findings are outlined and discussed in the context of common anatomical concepts and the more recently described prosomeric, segmental, model of brain development.

## Materials and Methods

### Ethics Statement

Experimental procedures were approved by the Regional Ethics Committee No. 3 of Ile-de-France region on Animal Experiments and followed the guidelines of the European Communities Council Directive (86/809/EEC) and the Ministère de l’Agriculture et de la Forêt, Service Vétérinaire de la Santé et de la Protection Animale (Permit No. A 94-028-21).

### Embryo Section Preparation

**FIGURE 1:**
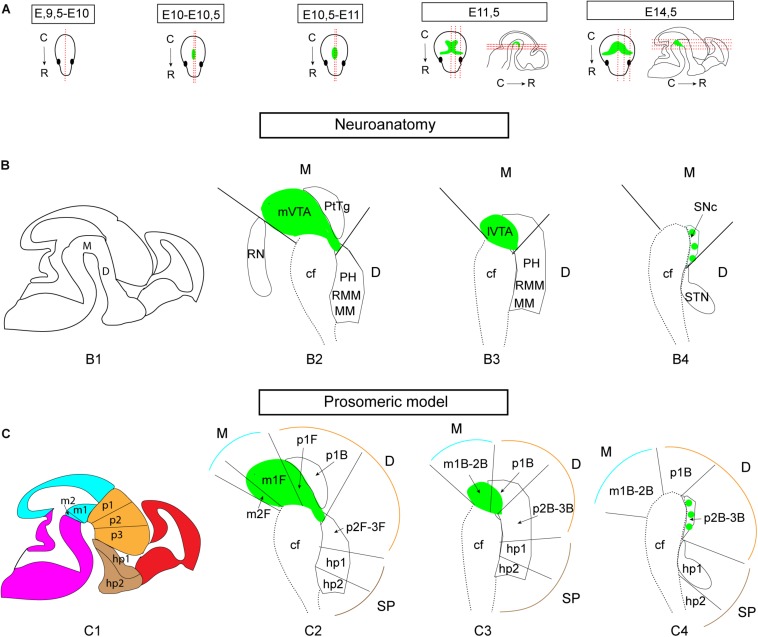
Illustrations of the developing mouse brain to visualize sectioning angles and anatomical and neuromeric nomenclature implemented in the study. The midbrain DA system is shown in green for orientation, based on Th mRNA labeling. **(A)** Sagittal and horizontal sectioning angles of the brain at the different developmental stages used in the current study (E9.5-E10, E10-10.5, E10.5-E11, E11.5, and E14.5). **(B,C)** Schematic sagittal representations of three different medio-lateral levels of the brain of a E14.5 embryo **(B1,C1)** in which the mes-di-encephalic areas are shown in close-ups **(B2–B4,C2–C4)** to visualize the neuroanatomical **(B)** and prosomeric **(C)** terminology. The VTA is present in mesomers m1, m2, and prosomere (p) 1; the SNc is present in p2 and p3. Original drawings achieved by the use of published brain atlases (http://developingmouse.brain-map.org) and recent literature in which the medial section level is presented (Puelles and Rubenstein, 2003, 2015; Marín et al., 2005; Thompson et al., 2014; Puelles, 2019). Note that the VTA and SNc are part of the mesencephlon (M, blue) in standard anatomical terminology based on the columnar model, but part of both the M and diencephalon (D, orange) in the prosomeric model due to a change in the position of the border between M and D (Puelles, 2019). E, embryonic day; C, caudal; cf, cephahlic flexure; hp, hypothalamo-telencephalic prosomer; m, mesomer (mesencephalic prosomer); MM, mammillary nucleus; p, diencephalic prosomer; PH, posterior hypothalamus; PtTg, pretectal tegmentum; R, rostral; RMM, retromammillary nucleus; RN, raphe nucleus; SNc, substantia nigra *pars compacta*; STN, subthalamic nucleus; VTA, ventral tegmental area; SP, pallium/subpallium. (B) Refers to basal plate origin. F refers to floor plate origin.

Mice were mated and females checked for vaginal plug in the morning. For determination of embryo stage, morning of vaginal plug was considered as embryonal day (E) 0.5. embryos were collected at E 9.5, E10.5, E11.5, and E14.5. Pregnant females were euthanized by cervical dislocation and embryos removed and rapidly frozen in cold isopentane (−20°/−25°C). To investigate gene expression patterns, multiple brain sections were prepared and analyzed by *in situ* hybridization using 2 differently fluorophore-labeled probes per section. The same technique was used throughout the study in order enable direct comparisons between mRNA patterns. As all mRNAs are found in the cytoplasm, while proteins show different subcellular localization depending of functional role, by addressing mRNA rather than protein, co-localization of gene expression is possible to analyze. Throughout the study, several brains from embryos at the same embryonal stage have been serially sectioned (3 series for E9.5 to E11.5; 5 series for E14,5) on cryostat at 16 μm thickness to allow systematic analysis throughout rostro-caudal and dorso-ventral axes. Multiple sections have been processed by *in situ* hybridization, and representative examples are displayed in each figure. A schematic illustration of section angles provided for each developmental stage analyzed is illustrated in Figure 1.

### Double-Probe Fluorescent *in situ* Hybridization

#### DNA Template for Riboprobe Synthesis

DNA template with T3 and T7 promoter sequences on both sides of each cDNA target were produced by PCR using promoter-attached primers.

#### Probes

Double-probe fluorescent *in situ* hybridization (sdFISH) was performed using antisense riboprobes for the detection of the following mRNAs: Th NM_009377.1 sequence 456-1453; Slc17a6 (Vglut2) NM_080853.3 sequence: 2315-3244; slc17a6 (Vmat2) NM_0130331.1 sequence 701-1439; slc32a1 (Viaat) NM_009508.2 sequence 649-1488; Tph2 NM_173391.3 sequence 277-1262; Pitx2 NM_001042504.2 sequence 792-1579; Nr4a2 (Nurr1) NM_013613.2 sequence 257-984; Snca NM_019169.2 sequence 59-1037; Foxa1 NM_008259.3 sequence 403-1181; Otx2 NM_001286481.1 sequence 851-1633; Kcnj6 (Girk2) NM_010606.2 sequence 275-1025; Calbindin2 (Calb2) NM_007586.1 sequence 80-793; NeuroD6 NM_009717.2 sequence 635-1419; Calbindin1 (Calb1) NM_009788.4 sequence 79-870; Pitx3 NM_008852.4 sequence 629-1329; TrpV1 NM_001001445.2 sequence 426-1239; Fst NM_001301373.1 sequence 460-1221; Tacr3 NM_021382.6 sequence 421-1427; Grp NM_175012.3 sequence 134-858; Lpl NM_008509.2 sequence 680-1460; Ntf3 NM_001164034.1 sequence 538–1294. Synthesis of digoxigenin and fluorescein-labeled RNA probes were made by a transcriptional reaction with incorporation of digoxigenin or fluorescein labeled nucleotides (Sigma-Aldrich; Reference 11277073910 and 11685619910). Information of primer sequences for riboprobe synthesis in Table 1. Specificity of probes was verified using NCBI blast.

**TABLE 1:** Information of primer sequences implemented to synthesize mRNA-directed riboprobes for fluorescent in situ hybridization analysis.

**Riboprobe**		

**mRNA**	**5′ primer**	**3′ primer**
Fst	AATTAACCCTCACTAAAGGGAGCTGGCTCCGCCAAGCAAAG	TAATACGACTCACTATAGGGTGGCACAGACCGGCTCATCC
Tacr3	AATTAACCCTCACTAAAGGGAGCCTCCGTGGCTGCCTTCAA	TAATACGACTCACTATAGGGCAGGCTGCTCTGCCGTGTGG
Grp	AATTAACCCTCACTAAAGGGACGGCTCGGAGCTCTCGCTCT	TAATACGACTCACTATAGGGGAATGGTAGCAAATTGGAGCCCTGA
Lpl	AATTAACCCTCACTAAAGGGAGAGCCCATGCTGCTGGCGTA	TAATACGACTCACTATAGGGCACCAGTCGGGCCAGCTGAA
Ntf3	AATTAACCCTCACTAAAGGGAGGCACCCAGGGAACCAGAGC	TAATACGACTCACTATAGGGGGCCTGAGGGAAGGCAAGCA
Neurod6	AATTAACCCTCACTAAAGGGAACCGGATGCACGGCCTCAAT	TAATACGACTCACTATAGGGTGCCAATTACGCAGCCCACA
Calb2	AATTAACCCTCACTAAAGGGACGCAGCAGCAGCCCCCTTAC	TAATACGACTCACTATAGGGTGGTGAGCTGTTGGATGTTCATCTCC
Trpv1	AATTAACCCTCACTAAAGGGAGCGCCTGACTGACAGCGAGT	TAATACGACTCACTATAGGGCATGTCGTGGCGGTTGGGGG
Snca	AATTAACCCTCACTAAAGGGATCAGAAGCCTAGGGAGCCGTGT	TAATACGACTCACTATAGGGAGACAGAAAGAGTCATTCAGGACACCT
Viaat	AATTAACCCTCACTAAAGGGAGCCAGGGCCTGCAGATGGAC	TAATACGACTCACTATAGGGTCGCTGGGCTGCTGCATGTT
Vglut2	AATTAACCCTCACTAAAGGGACCTTGGGCAGACCCTGAGGAA	TAATACGACTCACTATAGGGGGGGGAGCATGGAGCATACCC
Vmat2	AATTAACCCTCACTAAAGGGATCCGTGGCTGGGATGGGTATG	TAATACGACTCACTATAGGGCCTTTGCGATGGCACCACCA
Otx2	AATTAACCCTCACTAAAGGGACCCAACCATTGCCAGCAGCA	TAATACGACTCACTATAGGGGCCATGACCTTCCCTCCCTTCC
Girk2	AATTAACCCTCACTAAAGGGAGAACCGGCGAGTCGGAGCTG	TAATACGACTCACTATAGGGCGGGGCACTTGTCCGTGATG
Pitx3	AATTAACCCTCACTAAAGGGAGCTCGCCGCCAAGACCTTCC	TAATACGACTCACTATAGGGGGGAGTCTGGAGAAGGCGGGG
Pitx2	AATTAACCCTCACTAAAGGGAGCGATGGTGGCCAGGCTAGG	TAATACGACTCACTATAGGGTCCTTTGCTCGCAAGCGAAAAATC
Tph2	AATTAACCCTCACTAAAGGGACAAAGAGCCCGGCAAAGGCG	TAATACGACTCACTATAGGGCTGCTCCATACGCCCGCAGT
Nurr1	AATTAACCCTCACTAAAGGGAGGGCTCCTCTGCTCCCGGAG	TAATACGACTCACTATAGGGATGCCGGCTTGCGAATGGGG
Foxa1	AATTAACCCTCACTAAAGGGAAGATGGAAGGGCATGAGAGCAACG	TAATACGACTCACTATAGGGCTGGCTTGTCCGGGGATCGT
Th	AATTAACCCTCACTAAAGGGAGTGCGTCGGGTGTCTGACGA	TAATACGACTCACTATAGGGTCCAAGGAGCGCTGGATGGTG
Gad2	AATTAACCCTCACTAAAGGGAGAACCCGGGCACAGCGAGAG	TAATACGACTCACTATAGGGACGCGATGAGCCTGGGCACT
Gad1	AATTAACCCTCACTAAAGGGACACGCCTTCGCCTGCAACCT	TAATACGACTCACTATAGGGGGTGACCTGTGCGAACCCCG
Calb1	AATTAACCCTCACTAAAGGGAAGCCCTCTCGCCCGAGGTTC	TAATACGACTCACTATAGGGCCCTCCATCCGACAAGGCCATTA

#### Procedure

Horizontal and sagittal sections were prepared on cryostat in series of 3 or 5 to encompass several markers on adjacent sections. All sections within the mes-di-encephalic area were analyzed in a minimum of two embryos per developmental stage and per mRNA. Cryosections were air-dried, fixed in 4% paraformaldehyde and acetylated in 0.25% acetic anhydride/100 mM triethanolamine (pH 8) followed by washes in PBS. Sections were hybridized for 18h at 65°C in 100 μl of formamide-buffer containing 1 μg/ml DIG-labeled riboprobe and 1 μg/ml fluorescein-labeled riboprobe. Sections were washed at 65°C with SSC buffers of decreasing strength, and blocked with 20% FBS and 1% blocking solution. For revelation steps, DIG epitopes were detected with HRP anti-DIG fab fragments at 1:2500 (Sigma-Aldrich; Reference 11207733910) and revealed using Cy3-tyramide at 1:100. Fluorescein epitopes were detected with HRP anti-fluorescein fab fragments at 1:5000 (Sigma-Aldrich; Reference 11426346910) and revealed using Cy2-tyramide at 1:250. Cy2-tyramide and Cy3-tyramide were synthetized as previously described (Hopman et al., 1998). Nuclear staining was performed with DAPI. All slides were scanned at 20x resolution using the NanoZoomer 2.0-HT (Hamamatsu, Japan). Laser intensity and time of acquisition was set separately for each riboprobe.

#### Data Analysis

Image analysis was performed using the ndp2.view software (Hamamatsu). A summary of all mRNAs analyzed and their detection at E11.5 and E14.5 is shown in Table 2.

**TABLE 2:** Summary of mRNAs analyzed at E11.5 and E14.5 and indication (Yes/No) if detected.

**mRNA analyzed by fluorescent *in situ* hybridization**	**Ventral midbrain mRNA detection**	
**mRNA identified at P3 in Viereckel et al., 2016 and analyzed in current study**	**E11.5**	**E14.5**
Fst	No	No
Tacr3	No	No
Grp	No	No
Lpl	No	No
Ntf3	No	No
Neurod6	No	Yes
Calb2	No	Yes
Trpv1	No	Yes
**Additional mRNA**	**E11.5**	**E14.5**
Snca	Yes	Yes
**v-transporters in ventral midbrain**	**E11.5**	**E14.5**
Viaat	Yes	Yes
Vglut2	Yes	Yes
Vmat2	Yes	Yes
**Reference mRNA in ventral midbrain or at borders**	**E11.5**	**E14.5**
Otx2	Yes	Yes
Girk2	No	Yes
Pitx3	Yes	Yes
Pitx2	Yes	Yes
Tph2	Yes	Yes
Nurr1	Yes	Yes
Foxa1	Yes	Yes
Th	Yes	Yes
Gad2	No	Yes
Gad1	No	No
Calb1	No	Yes

*v-transporters; vesicular neurotransmitter transporters.*

## Results

In comparison with the adult brain, in which the SNc and VTA can be subdivided into anatomical subareas (Fu et al., 2012), complete subdivisions can not be definitely distinguished during early brain development as some cells are still migrating to their final positions (Brignani and Pasterkamp, 2017). However, presumptive VTA and SNc DA neurons, some of which may have not reached their final positions yet, can be detected as distributed in medial-lateral manner. To visualize the entire mDA area during early brain development, serial sectioning at the horizontal and sagittal planes at multiple stages (E9.5-14.5) was performed to allow for careful histological analysis (Figure 1A). Further, while DA neurons of the VTA and SNc classically are considered to originate and reside in the midbrain, mesencephalon, a shift in interpretation of developmental morphology from the so called “columnar model” to the “prosomeric model” have placed the VTA and SNc in both the mesencephalon and diencephalon due to a narrowing of the mesencephalon (reviewed by Puelles, 2019). In the prosomeric model, the VTA corresponds to mesomeres (m) m1, m2 and prosomere (p) p1, while the SNc corresponds to p2-3 (Figures 1B,C). In the current study, both standard neuroanatomical concepts (Figure 1B) and nomenclature of the prosomeric model (Figure 1C) have been implemented for the purpose of clarity. For sake of coherence, developing DA neurons to be located in the VTA and SNc areas have been referred to as mDA neurons.

### Different Distribution of Vglut2, Viaat, and Vmat2 mRNAs in the Mes-Di-Encephalon at E14.5

**FIGURE 2:**
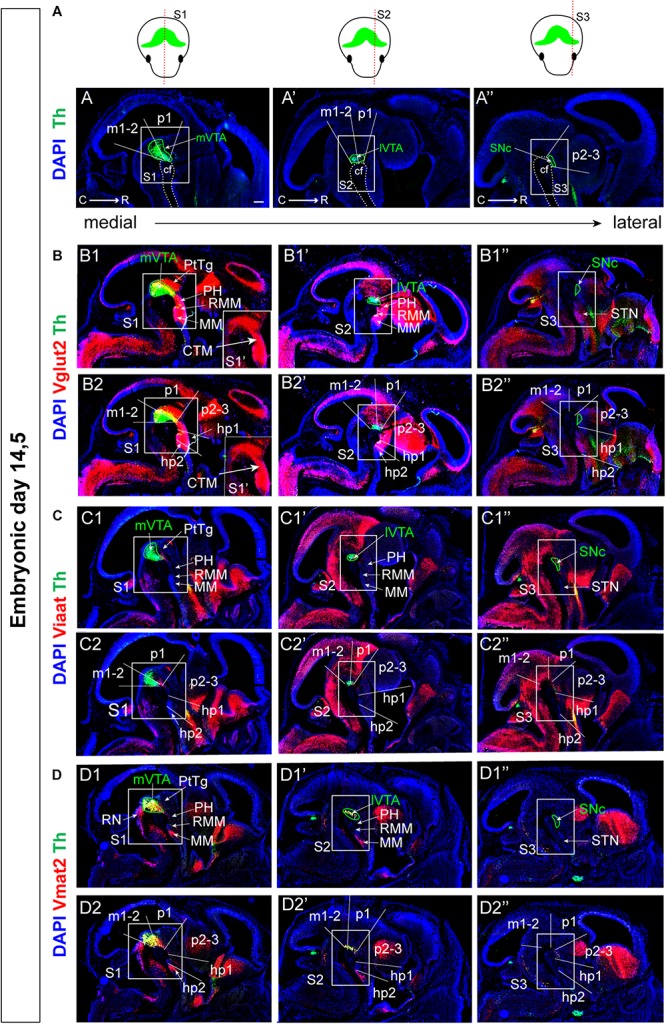
Different distribution patterns of vesicular transporters Vglut2, Viaat, and Vmat2 mRNAs in mes-and di-encephalic structures at E14.5. **(A–D)** Detection of mRNA for vesicular neurotransmitter transporters and their relation to Th mRNA in three medial-lateral sagittal levels (S1–S3; indicated in schematical illustration in top row) as detected by double-fluorescent *in situ* hybridization at E14.5. Th (green, **A–D**); in red: Vglut2 **(B)**; Viaat **(C)**; Vmat2 **(D)**. Squares (S1, S2, and S3) indicate the area within the mes-di-encephalic structure analyzed in the current study, encompasses parts of prosomeres m1, m2, p1, p2, p3, hp1, and hp1, and include the VTA, SNc, PH, RMM, and MM. Close-up in **B1** shows Vglut2 (red) mRNA only: Identification of a Vglut2-positive continuum (CTM) encompassing all the above listed prosomeres. **(B1,C1,D1)** Show neuroanatomical terms while **(B2,C2,D2)** show the same *in situ* hybridization results in the context of prosomeric terminology. Scale bars: **(A–D)**: 250 μm. C, caudal; cf, cephahlic flexure; CTM, continuum; hp, hypothalamo-telencephalic prosomer; m, mesomer (mesencephalic prosomer); MM; mammillary nucleus; p, diencephalic prosomer; PH, posterior hypothalamus; PtTg, pretectal tegmentum; R, rostral; RMM, retromammillary nucleus; SNc, substantia nigra *pars compacta*; STN, subthalamic nucleus; VTA, ventral tegmental area; lVTA, lateral VTA; and mVTA, medial VTA.

Vesicular neurotransmitter transporters (“v-transporters”) belong to the solute carrier family and enable pre-synaptic packaging of neurotransmitters for synaptic release; their presence thereby define a neuron’s neurotransmitter identity. VGLUT2 (encoded by *Vglut2*/*Slc17a6*) enables packaging of glutamate into presynaptic vesicles, VMAT2 (encoded by *Vmat2*/*Slc18a2*) packages monoamines, including DA, 5-HT, noradrenalin and adrenalin, and the vesicular inhibitory amino acid transporter VIAAT (aka VGAT, encoded by *Viaat/Vgat*/*Slc32a1*) packages GABA (Pupe and Wallén-Mackenzie, 2015). To address neurotransmitter identity during early brain development, the distribution of Vglut2, Vmat2, and Viaat mRNAs was analyzed. Based on the recent histological demonstration that the level of co-localization between Vglut2 mRNA and Th mRNA in mDA neurons is lower at E14.5 than in the newborn mouse (Papathanou et al., 2018), but can be detected also at E12.5 (Birgner et al., 2010), E14.5 and E11.5 were selected for initial analysis. At E14.5, sagittal sectioning enables visualization of mDA nuclei in several sections encompassing different medial-lateral positions, here represented as S1 (medial), S2 (more lateral) and S3 (lateral). Using a probe toward Th mRNA, DA neurons of the presumptive medial VTA (mVTA; prosomeres m1, m2, p1; section level S1), lateral VTA (lVTA; prosomeres m1, m2, p1; section level S2), and SNc (prosomeres p2, p3, section level S3) were labeled and identified (Figures 2A–A”).

On adjacent sections, Vglut2/Th, Viaat/Th, and Vmat2/Th double-labeling was performed showing that all v-transporters are expressed at this stage with individual distribution profiles (Figures 2B–D and Table 2). Sections were analyzed using neuroanatomical (Figures 2B1,C1,D1) and prosomeric (Figures 2B2,C2,D2) terminology. Vglut2 mRNA was detected throughout section levels S1-S3 in brain and spinal cord in cells lining the ventricular zones, i.e., in differentiating and differentiated cells (Figures 2B1–B1”,B2–B2”). Viaat mRNA was also broadly distributed, but with a stronger expression in lateral (S2, S3) than medial (S1) level and was excluded from cortical areas (Figures 2C1–C1”,C2–C2”). As expected, based on the restricted distribution of monoaminergic cells, Vmat2 mRNA was confined to monoaminergic areas of the ventral midbrain and hindbrain (Figures 2D1–D1”,D2–D2”). Both Vmat2 and Vglut2 co-localized with Th mRNA in the ventral mes-di-encephalon while Viaat appeared not to co-localize with Th at any section level (Figures 2B–D).

In contrast to the *Vmat2* gene, which was expressed in Th-positive neurons and in neurons caudally of the mVTA in the presumptive serotonergic (5′HT) raphe neurons (RN) of the rhombencephalon (Figures 2D1,D2), Vglut2 mRNA was not detected in all Th-positive neurons. Instead, Vglut2/Th co-localization was primarily found in the medial aspect of the VTA (mVTA) (Figures 2B1–B2”) confirming recent analysis performed at the same stage (Papathanou et al., 2018). Apart from mDA neurons, Vglut2 mRNA was prominent throughout the mes-di-encephalic area, although not equally strong labeling was seen in all cells positive for this glutamatergic marker. The area positive for Vglut2 mRNA formed a Vglut2-positive “continuum” from the caudal aspect of the VTA into the rostral diencephalon, primarily detected at the medial section level (Figures 2B1,B2). Within the medial mes-diencephalon, the Vglut2-positive continuum included the VTA, pretectal tegmentum (PtTg), posterior hypothalamus (PH), retromammillary nucleus (RMM), and mammillary nucleus (MM), encompassing the medial aspect of prosomeres m1, m2 of the mesencephlon, and p1, p2, p3, hp1, and hp2 of the diencephalon (Figures 2B1,B2). In these areas, multiple glutamatergic neurons reside in the mature brain. Vglut2 mRNA was less prominent in the mes-di-encephalon in lateral sections and was absent from the area of the developing SNc in lateral prosomer p2-3 and only weak in lateral hp1, hp2 (Figures 2B1’–B2”).

### Specification of a Vglut2-Positive Mes-Di-Encephalic Continuum and Rare Presence of Viaat in the Dopaminergic Area at E14.5

**FIGURE 3:**
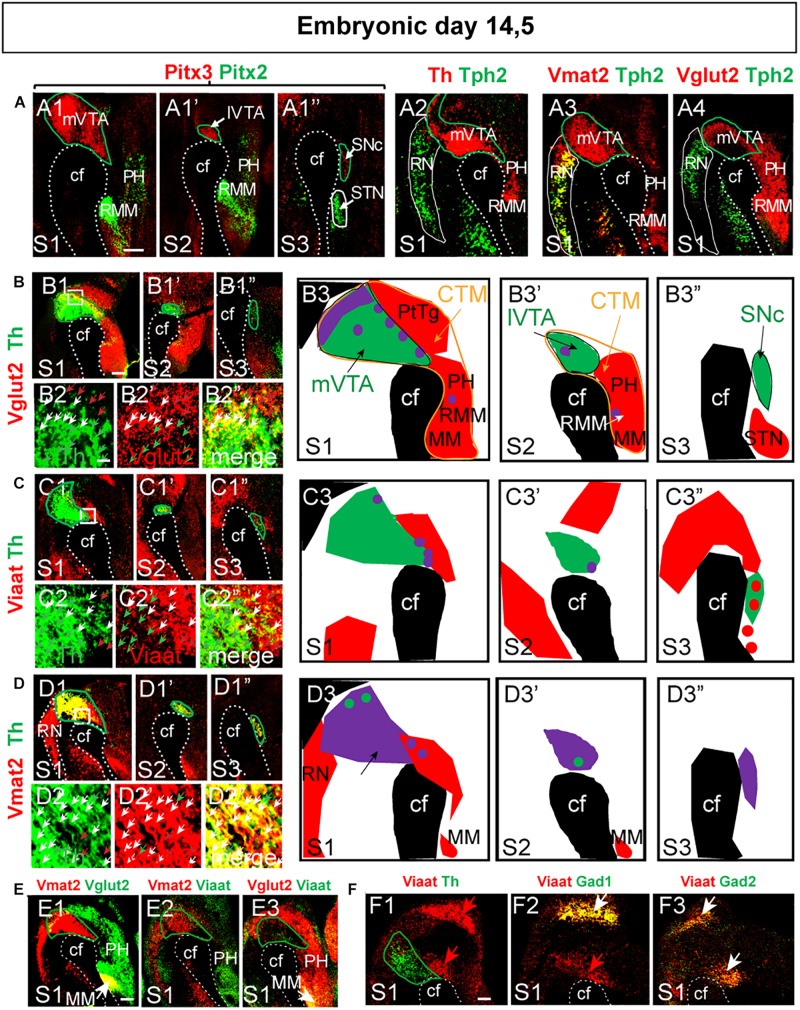
Identification of distinct patterns for vesicular transporters Vglut2, Viaat and Vmat2 mRNAs in mes-and di-encephalic structures at E14.5. Close-up:s of double-fluorescent *in situ* hybridization analysis within the mes-di-encephalic area encompassing parts of prosomers m2-hp2 in S1-S3 section levels, same sections and indications as outlined in Figure 2. **(A)** Borders between brain areas at S1–S3 levels identified by combinations of molecular markers detected at mRNA level by: Pitx3 (red)/Pitx2 (green) **(A1–A1”)**; Th (red)/Tph2 (green) **(A2)**; Vmat2 (red)/Tph2 (green) **(A3)**; and Vglut2 (red)/Tph2 (green) **(A4)**. **(B–D)** Structures lining the cephalic flexure **(B1,D1,E1)** with high-magnification images **(B2,C2,D2)** to pin-point discrete neurons: Vglut2 (red)/Th (green) **(B)**; Viaat (red)/Th (green) **(C)**; Vmat2 (red)/Th (green) **(D)**. Arrows in **(B2,C2,D2)** panels indicate double-positive cells. **(B3–C3)** Schematic illustration of Th mRNA (green) in combination with Vglut2 (red) **(B3)**, Viaat (red) **(C3)**, Vmat2 (red) **(D3)** mRNAs with co-labeled mRNAs illustrated in purple. **(E)** Relation between mRNAs for vesicular transporters at the S1 level. Vmat2 (red)/Vglut2 (green) **(E1)**; Vmat2 (red)/Viaat (green) **(E2)**; and Vglut2 (red)/Viaat (green) **(E3)**. **(F)** Comparison of Viaat with Gad1 and Gad2 mRNAs, S1 level. Viaat (red)/Th (green) **(F1)**; Viaat (red)/Gad1 (green) **(F2)**; Viaat (red)/Gad2 (green) **(F3)**. Overlap between mRNAs in yellow (except in schematic illustration; purple). Scale bars: **(A)**: 125 μm **(B1–B1”,C1–C1”,D1–D1”)** 175 μm; **(B2–B2”,C2–C2”,D2–D2”)** 15 μm; **(E)** 175 μm; **(F)** 125 μm. cf, cephalic flexure; CTM, continuum; MM; mammillary nucleus; PH, posterior hypothalamus; PtTg, pretectal tegmentum; RMM retromammillary nucleus; RN, raphe nucleus; SNc, Substantia nigra *pars compacta*; STN, subthalamic nucleus; VTA, ventral tegmental area, mVTA, medial VTA; and lVTA, lateral VTA.

To pinpoint the medial mes-di-encephalic expression of the *Vglut2* gene, as well as the expression patterns of *Vmat2* and *Viaat*, additional mRNAs were analyzed to allow the definition of borders between the mDA areas and neighboring areas rostrally and caudally. PITX homedomain proteins subtypes 2 and 3 (PITX2 and PITX3) have been described as markers for distinct neurons in the mes- and di-encephalon. PITX2 is confined to subsets of diencephalic glutamatergic neurons, most notably the subthalamic nucleus (STN) and para-STN (pSTN) (Martin et al., 2004) while PITX3 is found in mDA neurons from approximately E11.5 (Smidt et al., 1997). Pitx2/Pitx3 mRNA co-labeling analysis at S1-S3 levels visualized their excluding patterns of expression, thus demonstrating the border between the mDA neurons and diencephalic PITX2-positive glutamatergic neurons (Figure 3A and Table 2). Pitx3-labeling was evident in the mVTA, lVTA and SNc, and Pitx2-labeling in the RMM and PH as well as in the STN and pSTN, all of which are of diencephalic identity (Figures 3A1–A1”). In the caudal aspect, Tryptophan hydoxylase 2 (Tph2) mRNA was used as marker for developing 5′HT neurons of the RN. Th/Tph2 co-labeling analysis showed their excluding expression, thus visualizing the border between the RN and caudal VTA, while Vmat2/Tph2 showed extensive co-localization within the RN thus visualizing 5-HT neurons (Figures 3A2–A4).

Having established these borders, the expression of v-transporters was analyzed in more detail. Vglut2 mRNA was strongest dorsally and rostrally of the Th-labeled cells with prominent Vglut2-labeling in the PtTg, PH, RMM and MM (Figures 3B1–B3”). Vglut2 mRNA showed some co-localization with Th within the mVTA, rare co-localization in the lVTA and none in the laterally positioned SNc (Figures 3B1–B3”). Viaat mRNA was present in the mes-di-encephalic area but almost excluded from the VTA and SNc areas with very few Viaat/Th co-labeled cells in the lVTA and mVTA (Figures 3C1–C3”). Vmat2 mRNA showed prominent co-labeling with Th throughout mVTA, lVTA and SNc at this stage, with few Th-neurons that were negative for Vmat2 mRNA (Figures 3D1–D3”). Vmat2-positive neurons in the RN were, as expected, negative for Th mRNA (Figures 3D1,D3).

Finally, the level of co-localization between v-transporters was addressed. Vmat2/Vglut2 co-labeling showed a modest amount of double-positive cells in the mVTA but was strong within the MM of the diencephalon (Figure 3E1). Vmat2/Viaat co-labeling revealed no double-positive cells at all (Figure 3E2), while Vglut2/Viaat double-positive cells were detected in the MM (Figure 3E3). Although Viaat mRNA was excluded from Th-positive neurons, it was of interest to compare its expression pattern within the developing midbrain with those of *Gad1* and *Gad2*, which encode for GAD67 and GAD65, respectively, and which are often used as markers for GABA-signaling neurons. Within the developing midbrain, Gad1 and Gad 2 mRNAs were spatially restricted in a partly overlapping manner, however, Viaat mRNA showed the sum of Gad1 plus Gad2 mRNA-labeled cells, thus validating Viaat mRNA as marker for GABA neurons at this developmental stage (Figures 3F1–F3).

### A Vglut2-Positive Mes-Di-Encephalic Continuum Detected at E14.5 Is Detected Also at E11.5

**FIGURE 4:**
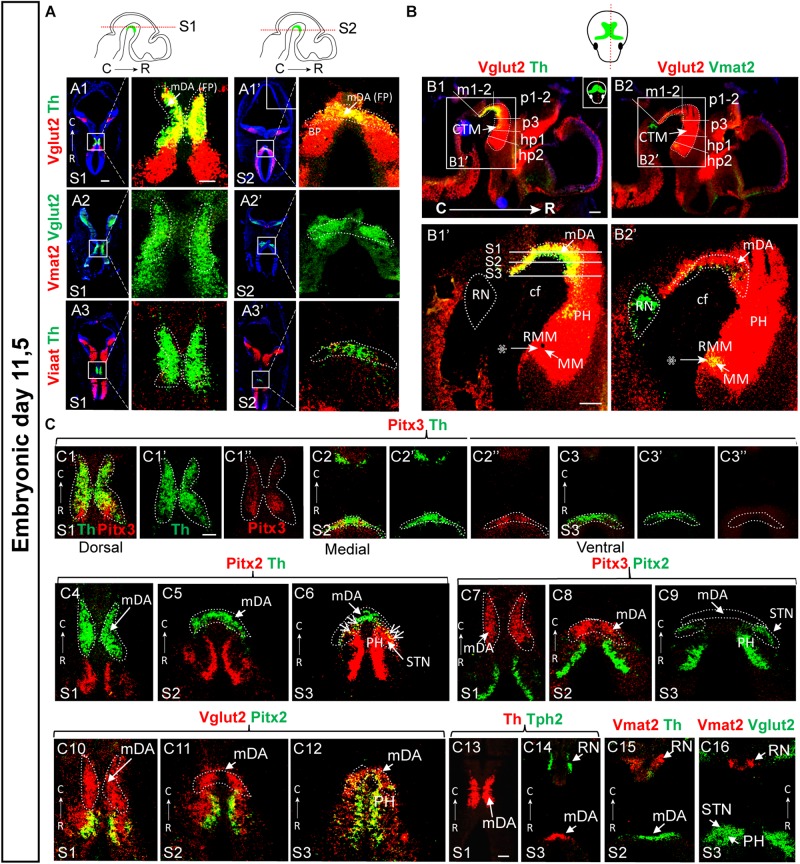
A Vglut2-positive mes-di-encephalic continuum encompassing parts of prosomeres m1 through to hp2 detected at E14.5 is observed also at E11.5. **(A,B)** Horizontal **(A)** and sagittal **(B)** sections of the embryonal mouse at E11.5, section angles as indicated in top panel, analyzed by double-fluorescent *in situ* hybridization. **(A)** Probes for detection of Vglut2 or Viaat mRNA co-labeled with probe for Th mRNA (for detection of mDA area) **(A1,A3)** or combining Vglut2 and Vmat2 probes **(A2)** in two ventral-dorsal horizontal levels (S1-S2); close-ups of Th/Vmat2-positive area to the right of each overview as indicated by squares: Vglut2 (red)/Th (green) **(A1,A1’)**; Vglut2 (green)/Vmat2 (red) **(A2,A2’)**; Viaat (red)/Th (green) **(A3,A3’)**. **(B)** Sagittal sections showing Vglut2 (red)/Th (green) **(B1,B1’)**; Vglut2 (red)/Vmat2 (green) **(B2,B2’)**. Stars point to Vglut2+/Vmat2+/Th- diencephalic neurons **(B1’,B2’)**. The Vglut2-positive (red) area, the Vglut2-continuum, is evident at this stage, and similar to E14.5 (Figures 2, 3) encompasses prosomeres m1, m2, p1, p2, p3, hp1, and hp2 with distinct borders caudally of m1 (border between RN and caudal VTA) and rostrally of hp2 (border rostral of MM). **(C)** Horizontal sections at three ventral-dorsal levels (S1–S3; depicted in **B1’**) showing mRNA for molecular marker mRNA serving to delineate boundaries between areas (used above to delineate borders). Pitx3 (red)/Th (green) **(C1–C3”’)**; Pitx2 (red)/Th (green) **(C4–C6)**; Pitx3 (red)/Pitx2 (green) **(C7–C9)**; Vglut2 (red)/Pitx2 (green) **(C10–C12)**; Th (red)/Tph (green) **(C13,C14)**; Vmat2 (red)/Th (green) **(C15)**; and Vmat2 (red)/Vglut2 (green) **(C16)**. Overlap between mRNAs in yellow. Scale bars **(A1–A3’)** 500 μm and close-ups 150 μm, **(B1–B2)** 250 μm, **(B1’–B2’)** 100 μm, **(C1–C12)** 150 μm, and **(C13–C16)** 250 μm. BP, basal plate; C, caudal; cf, cepahlic flexure; CTM, continuum; FP, floor plate; hp, hypothalamo-telencephalic prosomer; m, mesomer (mesencephalic prosomer); mDA, midbrain dopamine area; MM; mammillary nucleus; p, diencephalic prosomer; PH, posterior hypothalamus; R, rostral; RMM, retromammillary nucleus; RN, raphe nucleus; STN, subthalamic nucleus.

Based on the prominent expression of all three v-transporters at E14.5, including the observed mes-di-encephalic continuum of Vglut2-positive neurons, we next addressed the brain at a younger stage when DA progenitor cells start producing DA through their initiation of *Th* gene expression. E11.5 has been described as the first day of *Th* gene expression (Arenas et al., 2015) and with the translation into TH protein, *Th*-expressing neurons can synthesize DA. At this stage, no distinct subdivision of VTA and SNc exists, instead all DA neurons are located ventrally of the ventricular zone from which they subsequently migrate ventrally and tangentially to their final destinations (Hegarty et al., 2013; Arenas et al., 2015; Brignani and Pasterkamp, 2017; Smidt, 2017). Analysis of serial horizontal and sagittal sections of the developing mes and di-encephalon at E11.5 showed ample Vglut2 mRNA (Figures 4A1,A1’) and Viaat mRNA (Figures 4A3,A3’) in different distribution patterns while almost no Vmat2 mRNA at all was detected in this region at this stage (Figures 4A2,A2’). Vglut2/Th mRNA co-labeling showed substantial overlap at this stage, with significantly higher degree of co-expression than was observed at E14.5 (Figures 4A1,A1’). No, or very little, Viaat/Th co-expression was observed, instead Viaat was strong rostrally and caudally of the Th-positive area (Figures 4A3,A3’). Co-localization of Vglut2 mRNA with Th mRNA (Figures 4B1,B1’) and Vmat2 mRNA (Figures 4B2,B2’) showed that, similar as at E14.5, Vglut2 mRNA was prominent in the ventral midbrain and beyond this area rostrally, forming a continuum of Vglut2-positive cells. In the caudal aspect of this continuum, Vglut2 mRNA was strong immediately rostrally of the Vmat2 mRNA in the developing RN, but Vglut2 mRNA was excluded from the Vmat2-positive RN (Figures 4B2,B2’). Similar as at E14.5, Vmat2-positive 5′HT neurons of the RN thus form the caudal border of the Vglut2-expressing continuum which was detected throughout the mesencephalon (m1 and m2) through to the rostral diencephalon (p1, p2, p3, hp1, and hp2) including a rostrally located Vmat2-expressing cell group (Figures 4B2,B2’). Comparing Vmat2 mRNA within developing mDA neurons with that in the RN 5-HT neurons, Vmat2 expression at this early stage was more prominent in the RN than in the mDA area (Figures 4B2,B2’).

Next, Pitx2/Pitx3, Pitx2/Vglut2, Th/Tph2, Th/Vmat2, Vglut2/Vmat2 mRNA co-labelings in horizontal sections at 3 dorso-ventral levels were analyzed (illustrated as S1-S3 in sagittal view, Figure 4B1’). Pitx3/Th double-labeling showed that Pitx3 mRNA is most abundant in the rostral part of the Th-positive area at this stage, but that it is devoid from the most caudal Th-positive area (Figures 4C1–C1”,C2–C2”,C3–C3”). At E11.5, Pitx2/Th double labeling enabled the visualization of the border between developing DA neurons and the adjacent diencephalic Pitx2 neurons of the developing STN and pSTN (Figures 4C4–C6). Pitx2/Pitx3 double-labeling experiment again showed no overlap at all and verified the border between the developing dopaminergic and glutamatergic areas at E11.5 (Figures 4C7–C9). Further, most Pitx2-expressing STN neurons did not show detectable Vglut2 mRNA at this stage, instead only modest Pitx2/Vglut2 co-localization was detected (Figures 4C10–C12). Caudally, Tph2/Th double labeling showed no overlap at all, and thus enabled the definition of the border between dopaminergic and 5-HT neurons of the developing RN (Figures 4C13,C14). Finally, the horizontal angle verified the finding of the sagittal view above that Vmat2 mRNA was stronger in the RN than mDA area at this stage (Figure 4C15), while no RN neurons were positive for Vglut2 mRNA (Figure 4C16).

### Vglut2 Co-localizes With Nurr1 Throughout Most of the Mes-Di-Encephalic Continuum at E14.5

The mes-di-encephalic continuum of *Vglut2* expression was of interest to address further. NURR1/NR4A2 is a nuclear orphan receptor for which the mRNA can be found in the cephalic flexure from E10.5 in the mouse where it co-localizes with Th mRNA 1 day later (Zetterström et al., 1997). NURR1 has been demonstrated as crucial for mDA neuron differentiation and maintenance (Zetterström et al., 1997; Saucedo-Cardenas et al., 1998; Wallén et al., 1999; Kadkhodaei et al., 2009), and is commonly used to confirm a dopaminergic phenotype both *in vivo* and *in vitro*-generated neurons. Further, impairment of *Nurr1* expression has been implicated in the pathogenesis of PD, and *Nurr1* has been presented as a dysregulated target of α-synuclein toxicity (Decressac et al., 2013; Volakakis et al., 2015). To assess Vglut2 in mDA neurons further, endogenous localization of Nurr1 and Snca (encoding α-synuclein) mRNAs was addressed in relation to Vglut2 mRNA.

**FIGURE 5:**
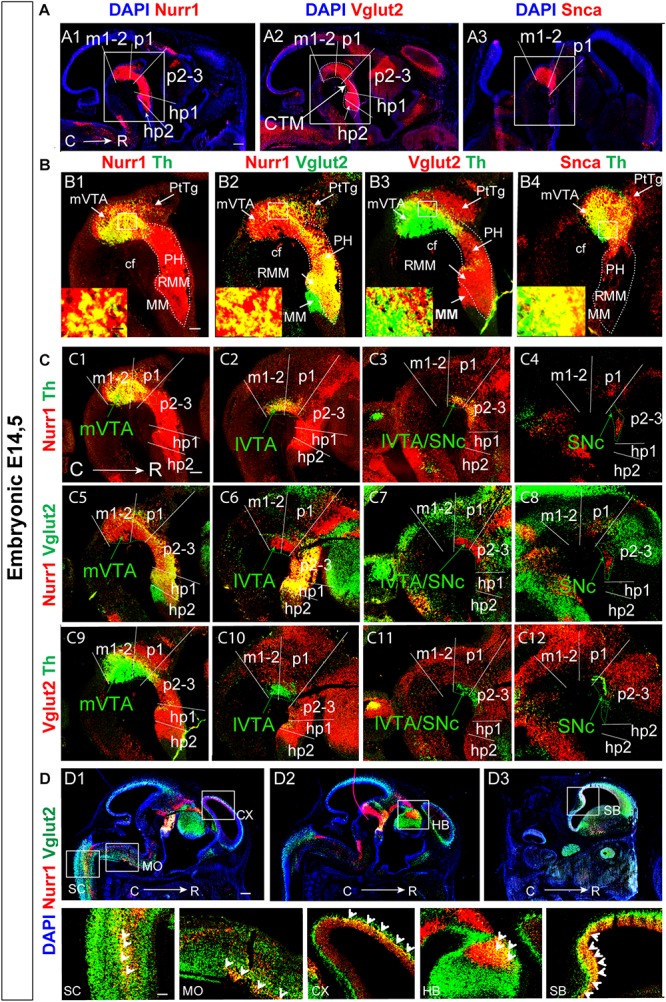
Vglut2 mRNA co-localizes with Nurr1 mRNA throughout most of the Vglut2-positive mes-di-encephalic continuum, and partially with Snca mRNA, at E14.5. Sagittal sections analyzed by double-fluorescent *in situ* hybridization in the E14.5 brain **(A–D)**. **(A)** In red, Nurr1 **(A1)**, Vglut2 **(A2)**, Snca **(A3)** mRNAs; DAPI counterstaining in blue. Squares indicate the mes-di-encephalic area encompassing prosomeres m1, m2, p1, p2, p3, hp1, hp2 at section level S1. **(B)** Co-labeling analysis of areas shown in squares in top panel: Nurr1 (red)/Th (green) **(B1)**; Nurr1 (red)/Vglut2 (green) **(B2)**; Vglut2 (red)/Th (green) **(B3)**; Snca (red)/Th (green) **(B4)**. High-magnification images in insets to show overlap on cellular level. **(C)** Co-labeling analysis at 4 different medial-lateral levels: Nurr1 (red)/Th (green) **(C1–C4)**; Nurr1 (red)/Vglut2 (green) **(C5–C8)**; Vglut2 (red)/Th (green) (C9-C12). **(D)** Nurr1 (red)/Vglut2 (green) in additional areas of the central nervous system show prominent overlap (yellow) in spinal cord (SC), medulla oblongata (MO), cortex (CX) **(D1)**, habenula (HB) **(D2)**, subiculum (SB) **(D3)** with close-ups in bottom panel. Scale bars **(A1–A3)** 250 μm, **(B1–B4)** 150 μm and close ups: 20 μm, **(C1–C12)** 150 μm, **(D1–D3)** 350 μm and close ups: 125 μm. cf, cephahlic flexure; hp, hypothalamo-telencephalic prosomer; m, mesomer (mesencephalic prosomer); MM; mammillary nucleus; p, diencephalic prosomer; PH, posterior hypothalamus; PtTg; pretectal tegmentum; R, rostral; RMM, retromammillary nucleus; SNc, substantia nigra *pars compacta*; VTA, ventral tegmental area; lVTA, lateral VTA; mVTA, medial VTA. Additional data presented in Supplementary Figure S1.

Sagittal sections at E14.5, and horizontal as well as sagittal sections at E11.5, were used to assess the level of co-localization of Vglut2 with Nurr1 mRNA and Snca mRNA in mDA neurons (Table 2, Figure 5, Supplementary Figure S1). When addressing medial sections at E14.5, it was immediately apparent that Nurr1 mRNA had a similar extensive expression in the mes-di-encephalon as Vglut2 mRNA, thus forming a Nurr1-positive continuum encompassing a similar neuromeric extension as Vglut2 (Figures 5A1,A2). With Nurr1 and Vglut2 positive extensions through m1, m2, p1, p2-3, hp1, a difference was detected in hp2 in which Nurr1 mRNA was lower or absent (Figures 5A1,A2). In contrast to the vast Nurr1 extension, Snca mRNA was primarily found in the m1, m2, p1 in which it showed a similar distribution as Nurr1 mRNA (Figure 5A3).

Nurr1/Th mRNA co-labeling confirmed previous studies that all Th-positive cells express the *Nurr1* gene within the midbrain (Figure 5B1). Vglut2/Nurr1 mRNA double-labeling in the mes-di-encephalon visualized abundant, but not complete, overlap between these two markers. By comparing with Vglut2/Th double-labeling, it was apparent that scattered Vglut2/Nurr1 co-labeled neurons were indeed detected in the mVTA dopaminergic area, but the strongest level of co-labeling was found in the PH and RMM areas and in surrounding neurons of the medial diencephalon (Figures 5B2,B3). Vglut2 was equally strong throughout the PtTg, PH, RMM, and MM areas (Figures 5A2,B2,B3), all of which have a strong glutamatergic neurotransmitter phenotype in the mature brain. Nurr1 was equally strong through the mVTA, PH, and RMM but the MM and PtTg were almost devoid of Nurr1 mRNA (Figures 5A1,B1,B2). Thus, no Vglut2/Nurr1 co-labeling was detected in either the MM or PtTg which were only positive for Vglut2 mRNA (Figure 5B2). In the ventral m1-2 area, Nurr1 was stronger than Vglut2 within the mVTA (Figures 5A1,A2,B2). Within the medial aspect of the mes-di-encephalon, Nurr1 is thus not restricted to Th-expressing DA neurons, in accordance with previous findings (Smits et al., 2013). Instead, Nurr1 mRNA is abundant in medially positioned mes-di-encephalic glutamatergic neurons.

In contrast to the strong detection of both Vglut2 and Nurr1 mRNAs in the medial aspect of the rostral diencephalon, only rare Snca-positive neurons were observed in the PH and RMM (Figure 5B4). Snca mRNA co-localized extensively with Th mRNA in the mVTA dopaminergic area, but some Th-positive neurons devoid of Snca were identified in the ventral aspect of the mVTA, and not all Snca-positive neurons had detectable Th mRNA (Figure 5B4).

By analysis of multiple serial sections covering the medial to lateral aspect of the mes-di-encephalon at E14.5 (Figures 5C1–C12), it was even more apparent that less Vglut2 mRNA was detected in laterally than medially positioned Nurr1-positive and Th-positive neurons. As described above, dopaminergic neurons of the SNc were devoid of Vglut2 mRNA, and by multiple-section analysis, it was clear that the lateral-most aspect of the mVTA as well as the lVTA contained very few, if any, Vglut2-positive dopaminergic neurons at this stage (Figures 5C5–C12). In accordance with literature, Nurr1 mRNA was equally strong at all medial-lateral levels throughout the mDA Th-positive area (Figures 5C1–C8).

Finally, in addition to Vglut2/Nurr1 co-localization in differentiating mDA and glutamatergic neurons in the mes-di-encephalon, substantial Vglut2/Nurr1 co-labeling was detected in the spinal cord, medulla oblongata, cerebral cortex, habenula and septum at E14.5, areas that all contain glutamatergic neurons in the mature mouse (Figures 5D1–D3).

### Vglut2 Co-localizes Extensively With Nurr1 and Snca at E11.5

**FIGURE 6:**
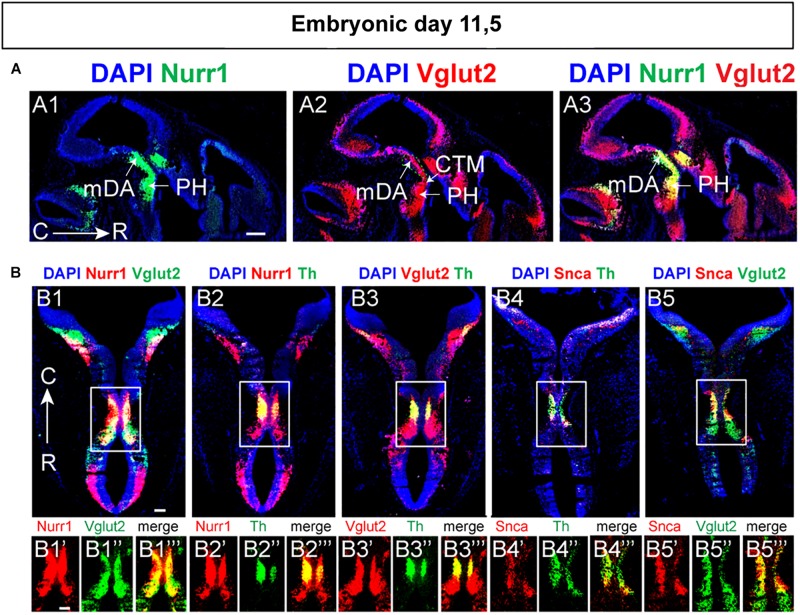
Substantial co-localization of Vglut2 mRNA with Nurr1 and Snca mRNAs observed in the mes-di-encephalic area at E11.5. Horizontal sections analyzed by double-fluorescent *in situ* hybridization in the E11.5 mouse brain. **(A)** In green, Nurr1 **(A1)**; in red Vglut2 **(A2)** and co-labeling Vglut2 (red)/Nurr1 (green) **(A3)**. **(B)** Co-labeling combinations at mesencephalic level Nurr1 (red)/Vglut2 (green) (**B1**; close-up **B1’–B1”’**), Nurr1 (red)/Th (green) (**B2**; close-up **B2’–B2”’**), Vglut2 (red)/Th (**B3**; close-up **B3’–B3”’**), Snca (red)/Th (green) (**B4**; close-up **B4’–B4”’**), and Snca (red)/Vglut2 (green) (**B5**; close-up **B5’–D5”’**). Sections counterstained with DAPI. Overlap between mRNAs in yellow. Scale bars **(A1–A3)** 250 μm and **(B)** 150 μm.

Next, E11.5 was addressed which confirmed substantial Vglut2/Nurr1 mRNA co-labeling in the medial mes-di-encephalon also at this stage (Figures 6A1–A3,B1–B1”’). In contrast to the absence of Vglut2/Th and Vglut2/Nurr1 co-labeling in lateral sections of the mDA area at E14.5, co-labeling analysis at E11.5 showed that both Nurr1 and Vglut2 co-localized extensively with Th throughout the mDA area at this stage (Figures 6B2–B2”’,B3–B3”’). In addition, both Nurr1 and Vglut2 mRNA were also found outside the mDA area, and co-localized rostrally of the Th mRNA-positive mDA area as well as in the hindbrain (Figures 6B1–B3”’). Also Snca mRNA was detected in the Th-positive mDA area at E11.5 and co-localized substantially with both Th and Vglut2 mRNA at this developmental stage (Figures 6B4–B5”’). Summarizing, at E11.5, Snca mRNA is found in a restricted number of Th-positive neurons, but both Vglut2 and Nurr1 mRNAs are detectable in a medial continuum along the cephalic flexure where they co-localize extensively albeit not completely. Further, while Nurr1/Th co-labeling is prominent in mDA neurons at all medial-lateral levels at both E11.5 and E14.5, Vglut2 co-labeling with both Th and Nurr1 in the mDA area is more pronounced at E11.5 than E14.5.

### Vglut2 mRNA Detected in Mes-Di-Encephalon Already at E9.5 Followed by Nurr1 and Th

**FIGURE 7:**
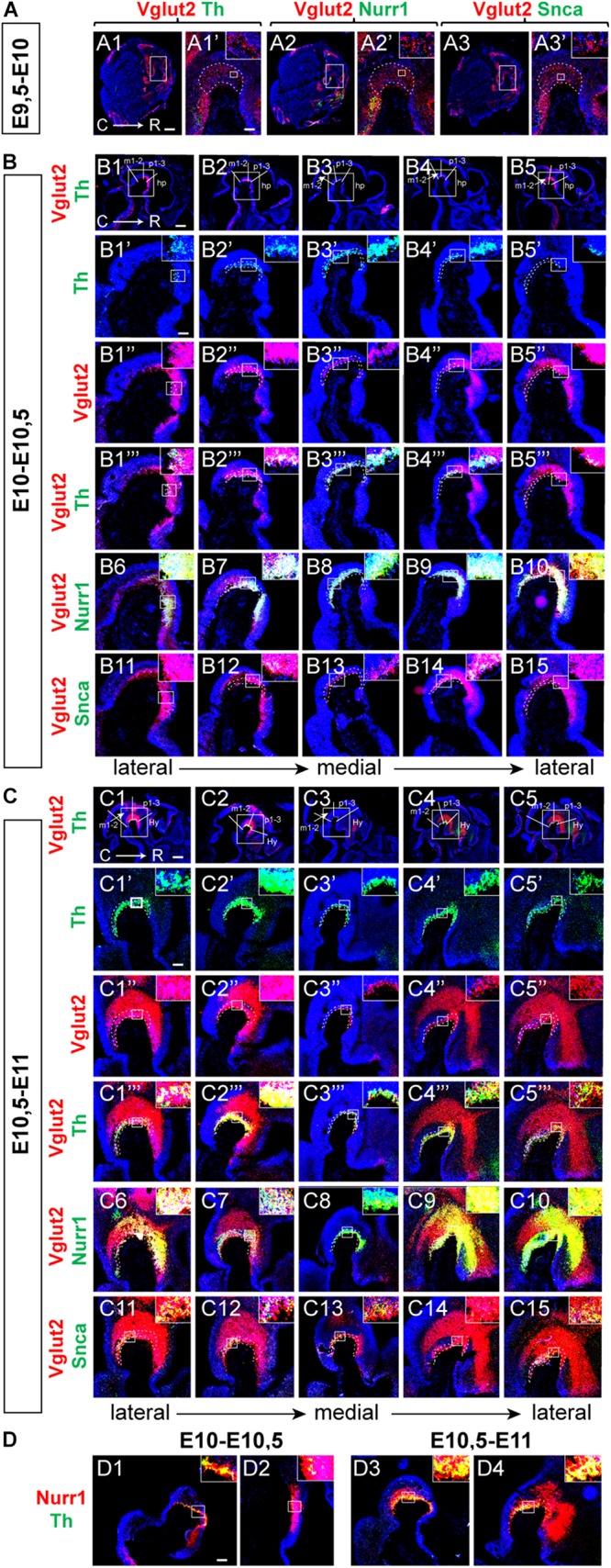
Vglut2 mRNA detected in mes-diencephalon already at E9.5 followed by Nurr1 and Th mRNAs. Sagittal sections analyzed by double-fluorescent *in situ* hybridization in the mouse brain at multiple stages to ascertain early developmental time-line. **(A–C)** Analysis at E9.5-10 **(A)**, E10-10.5 **(B)**, E10.5-11 **(C)** in showing Vglut2 (red)/Th (green) **(A1–A1’, B1–B5,B1”’–B5”’,C1–C5,C1”’–C5”’)**; Vglut2 (red)/Nurr1 (green) **(A2–A2’,B6–B10,C6–C10)**; Vglut2 (red)/Snca (green) **(A3–A3’,B11–B15, C11–C15)**. Th (green) **(B1’–B5’,C1’–C5’)**; Vglut2 (red) **(B1”–B5”,C1”–C5”)**. Close-ups in inset. **(D)** Nurr1 (red)/Th (green) at E10-10.5 **(D1–D2)**; E10.5-E11 **(D3–D4)**. Counterstaining with DAPI (blue). Red/green overlap shown in yellow. C, caudal; R rostral. Scale bars **(A1–A3)** 600 μm, **(A1’–A3’)** 125 μm, and **(B1–B5)** 500 μm. **(B1’–B15)** 125 μm, **(C1–C5)** 500 μm, **(C1’–C15)** 125 μm, and **(D1–D4)** 125 μm.

The extensive overlap between Vglut2 and Nurr1 mRNAs, and their co-labeling with Snca at E11.5 and E14.5, was of interest to address during earlier developmental stages to find out their temporal order. Vglut2/Th, Vglut2/Nurr1, Vglut2/Snca probe combinations were analyzed in brains of embryos dissected at E9.5 and E10.5. Because time post-coitum is not exact, and because the embryos were of different size within each litter, embryos were analyzed in three groups based on their size and dissection date (E9.5-E10; E10-E10.5; E10.5-E11). In E9.5-E10 embryos, no or very little Th (Figure 7A1), Nurr1 (Figure 7A2) or Snca (Figure 7A3) mRNA was detected in the along the cephalic flexure, but both Nurr1 and Th was detected in the medulla, caudally of the flexure. In contrast to absence of Th, Nurr1 and Snca mRNA in the ventral mes-di-encephalon, Vglut2 mRNA was readily detected in this area already at E9.5-10 (Figures 7A1–A3’).

At E10-10.5, Vglut2-positive neurons were pronounced along the cephalic flexure, in a mes-di-encephalic continuum similar as described above (Figure 7B). Vglut2 co-localized substantially with both Th mRNA (Figures 7B1–B5”’) and Nurr1 mRNA (Figures 7B6–B10), both of which were substantially more prominent at this stage than the day before, while only rare Snca-positive neurons were detected (Figures 7B11–B15). Notably, most, or even all, Th-positive (Figures 7B1–B5”’) and Snca-positive (Figures 7B11–B15) cells in the midbrain contained detectable levels of Vglut2 mRNA at this stage. Similar as E11.5 and E14.5, Nurr1 showed a distribution that formed a Nurr1-positive continuum similar as the Vglut2-positive continuum already at E10-10.5, with strongest Vglut2/Nurr1 overlap in the diencephalon (Figures 7B6–B10).

At E10.5-11, the Vglut2-positive area was even more pronounced than earlier, with clearly distinct Th mRNA labeling in the ventral aspect of the cephalic flexure (Figures 7C1–C5”’). Most Th-positive neurons were positive also for Vglut2 mRNA, however, Th-positive/Vglut2-negative neurons could also be discerned at this stage (Figures 7C1–C5”). Prominent overlap between Vglut2 and Nurr1 was detected throughout the mes-di-encephalon, but it was evident that a substantial amount of neurons in the dorsal mesencephalon were positive for only for Vglut2 mRNA (Figures 7C6–C10). Vglut2/Nurr1 overlap at E10.5-11 was strongest in the ventral mesencephalon and in the diencephalon (Figures 7C6–C10). At this stage, expression of the *Snca* gene had been upregulated with clear Vglut2/Snca overlap (Figures 7C11–C15). Finally, Nurr1/Th mRNA overlap was also addressed (Figures 7D1–D4). There were substantially more Nurr1-positive than Th-positive neurons at both E10-E10.5 (Figures 7D1,D2) and E10.5-E11 (Figures 7D3,D4), however, all Th-positive neurons contained Nurr1. In accordance with the demonstration above of a Nurr1-positive mes-di-encephalic continuum, a substantial proportion of Nurr1-positive neurons were negative for Th mRNA.

**FIGURE 8:**
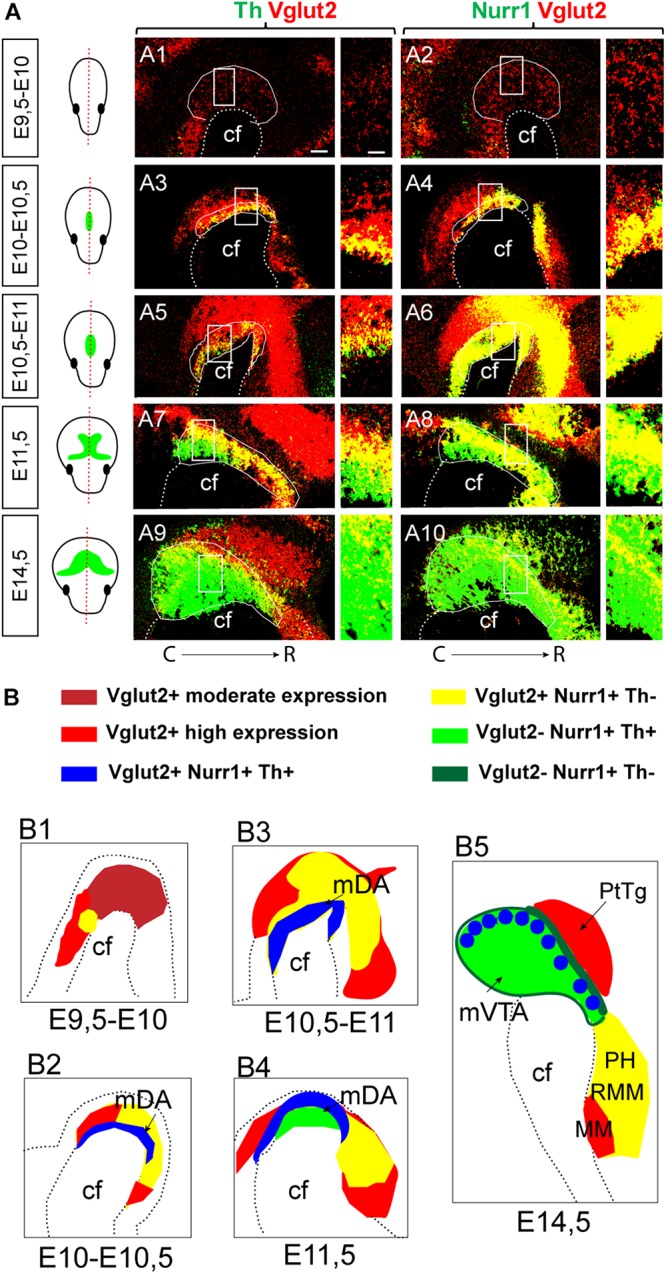
Summary of Vglut2, Nurr1 and Th mRNAs in the mes-and di-encephalon at E9.5-E14.5. Representative examples of sagittal sections at S1 section level analyzed by double-fluorescent *in situ* hybridization in the mouse brain at multiple stages; section level indicated in schematic illustrations. Green color in schematics illustrate the mDA system, outlined by Th mRNA labeling. **(A)** Co-labeling of Th (green)/Vglut2 (red) in left panel **(A1,A3,A5,A7,A9)**; Nurr1 (green)/Vglut2 (red) in right panel **(A2,A4,A6,A8,A10)**; close-ups of squares to the right. Co-localization of mRNAs in yellow. Stages: E9.5-10 **(A1–A2)**; E10-10.5 **(A3–A4)**; E10.5-11 **(A5–A6)**; E11.5 **(A7–A8)**; E14.5 **(A9–A10)**. **(B)** Schematic illustration of Vglut2, Nurr1, and Th mRNAs with overlap shown in different colors, see legend at top. Note Vglut2 mRNA detection of moderate and high level indicated by different shades of red. Stages: E9.5-10 **(B1)**; E10-10.5 **(B2)**; E10.5-11 **(B3)**; E11.5 **(B4)**; and E14.5 **(B5)**. Brain areas indicated: cf, cephalic flexure; mDA, midbrain dopamine neuron area; MM, mammillary nucleus; PH, posterior hypothalamus; PtTg, pretectal tegmentum; RMM retromammillary nucleus; VTA, ventral tegmental area. Caudal (C) to the left; Rostral (R) to the right in each picture. Scale bars **(A1–A10)** 100 μm and close ups: 50 μm.

By condensing the results of Vglut2/Th and Vglut2/Nurr1 co-labeling for each stage analyzed between E9.5 and E14.5, the time-line and spatial distribution of these mRNAs could be visualized with Vglut2 appearing first, detected already at E9.5, followed by Nurr1 and Th mRNAs (Figure 8A). Nurr1 shows substantial overlap with Vglut2 along a mes-di-encephalic continuum and seemingly all Th-neurons are initially positive for Vglut2 mRNA. However, Vglut2 mRNA is subsequently down-regulated in mDA neurons to mainly be found in the medial aspect of the VTA at E14.5. At this stage, the majority of Vglut2/Nurr1-positive cells are found in the rostral diencephalon, rather than in the area of the VTA (Figure 8A with schematic illustrations in Figure 8B). In addition to its expression in neurons destined to maintain a glutamatergic phenotype, Vglut2 mRNA thus seem to be present in all early differentiating DA neurons (E10-10.5), and is present prior to detectable levels of either Nurr1 or Th mRNA (E9.5-10).

**FIGURE 9:**
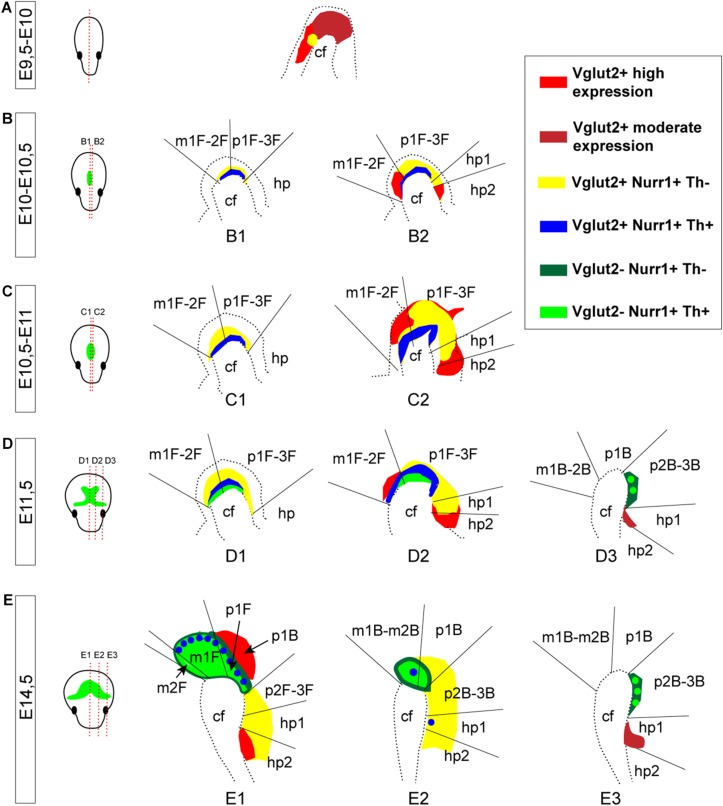
Summary of Vglut2, Nurr1, and Th mRNAs in prosomeric terminology representing different medial-to-lateral section levels of the mes-and di-encephalon at E9.5-E14.5. **(A–E)** Schematic illustration summarizing Vglut2, Nurr1, and Th mRNAs at each developmental stage analyzed with mRNA overlap represented by different colors, see legend at far right. Note Vglut2 mRNA detection of moderate and high level indicated by different shades of red. Section levels shown in illustration in far left panel (green color illustrate the mDA system, outlined by Th mRNA labeling). Stages: E9.5-10 **(A)**; E10-10.5 **(B)**; E10.5-11 **(C)**; E11.5 **(D)**; and E14.5 **(E)**. Caudal to the left; rostral to the right in each picture. cf, cephalic flexure; hp, hypothalamo-telencephalic prosomer; m, mesomer (mesencephalic prosomer); p, diencephalic prosomer. (B) Refers to basal plate, F refers to floor plate.

A schematic summary of the histological analysis of Vglut2, Nurr1, and Th mRNAs visualizes the extent of Vglut2/Nurr1 overlap and their overlap with Th mRNA across neuromers m1, m2, p1, p2, p3, and hp1 and also how the intensity of the labelings varies within these segments (Figure 9). The lack of Nurr1 in the Vglut2-positive medial aspect of hp2 (corresponding to MM) and medio-dorsal aspect of p1 (corresponding to PtTg) at E14.5 is evident already earlier during development, but apart from these areas, there is considerable overlap between Vglut2 and Nurr1 in the mes-di-encephalon already from E10 through to E14.5. Further, most, if not all, Th-positive neurons seem to express *Vglut2* prior to onset of Nurr1 and Th mRNAs (Figure 9).

### Time-Line of Midbrain Marker Appearance Indicates Early Diversity

With the finding that Nurr1 mRNA co-localizes with Vglut2 mRNA along the cephalic flexure already from E10, it was of interest to investigate the heterogeneity of this area in more detail. Using a non-biased microarray analysis to identify differential gene expression patterns throughout the VTA versus the SNc in the newborn mouse, we previously identified a number of genes, subsequently confirmed by histological validation at mRNA level to be expressed in discrete VTA neurons (Viereckel et al., 2016). These mRNAs included NeuroD6, Gastrin releasing peptide (Grp), Lipoprotein lipase (Lpl), Follistatin (Fst), Tachykinin receptor 3 (Tacr3), Neurotrophin 3 (Ntf3) and Transient receptor potential cation channel subfamily V member 1 (TrpV1), all of which co-localized with Th, while Ntf3 also co-localized with Viaat mRNA, and TrpV1 co-localized with both Vglut2 and Viaat mRNAs (Viereckel et al., 2016). Several of these mRNAs have been reported in microarray and scRNAseq analyses of mDA neurons, most notably Grp and NeuroD6 which have been identified in multiple gene expression analyses and to partially overlap with each other in the VTA (Chung et al., 2005; La Manno et al., 2016; Kramer et al., 2018). Based on our identification of their expression in the newborn mouse (Viereckel et al., 2016), these markers were next selected for analysis at E11.5 and E14.5 to determine the developmental time-line and distribution in the context of neurotransmitter phenotype. Fst, Tacr3, Grp, Lpl, Ntf3, NeuroD6, TrpV1 and Calbindin 2 (Calb2) mRNAs were analyzed in relation to Vglut2, Viaat, and Th at E11.5 and E14.5. Further, Otx2, Calb1, and Girk2 mRNAs were included as references based on their reported expression in the VTA (Otx2) and SNc (Calb1 and Girk2), and Foxa1 was included as reference based on its described role in initiation of DA neuron development (Veenvliet and Smidt, 2014; Arenas et al., 2015). At E14.5, also Snca mRNA was included to analyze its distribution at this stage.

**FIGURE 10:**
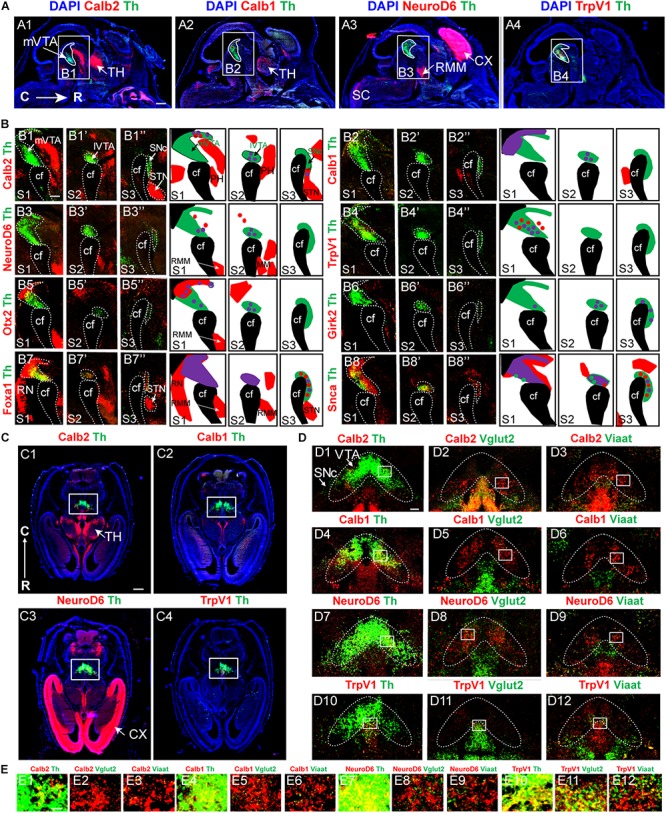
NeuroD6, Calb2, Calb1, and Trpv1 mRNAs are all detected in the mes-di-encephlon at E14.5 but not E11.5 in the developing mouse embryo. Sagittal **(A,B)** and horizontal **(C–E)** sections at E14.5. Additional data, see Table 2. **(A,B)** Overview of brain **(A)** with close-ups mes-di-encephlon in **(B)**. Calb2 (red)/Th (green) **(A1,B1–B1”)**, Calb1 (red)/Th (green) **(A2,B2–B2”)**, NeuroD6 (red)/Th (green) **(A3,B3–B3”)**, TrpV1 (red)/Th (green) **(A4,B4–B4”)**, Otx2 (red)/Th (green) **(B5–B5”)**, Girk2 (red)/Th (green) **(B6–B6”)**, Foxa1 (red)/Th (green) **(B7–B7”)**, and Snca (red)/Th (green) **(B8–B8”)**. Schematics to the right summarize findings with overlapping mRNAs illustrated in purple. **(C–E)** Calb2, Calb1, NeuroD6, TrpV1 mRNAs (all in red) co-analyzed with Th, Vglut2, Viaat (all in green). Close-ups on cellular level from squares in **(D)** are shown in panel **(E)**. Sections counterstained with DAPI (blue). Scale bars **(A)** 500 μm; **(B)** 250 μm; **(C)** 500 μm; **(D)** 100 μm; and **(E)** 20 μm. cf, cephalic flexure; CX, cortex; RMM, retromammillary nucleus; RN, raphe nucleus; STN, subthalamic nucleus; SNc, Substantia nigra *pars compacta*; TH, thalamus; VTA, ventral tegmental area, lVTA, lateral VTA; and mVTA, medial VTA.

At E11.5, none of Fst, Tacr3, Grp, Lpl, Ntf3, NeuroD6, TrpV1, Girk2, Calb2, or Calb1 mRNAs was detected in the mes-or di-encephalon (Table 2). At E14.5, Fst, Tacr3, Grp, Lpl, and Ntf3 mRNAs were still not detected (Table 2). However, Calb2, Calb1, Girk2, NeuroD6, and TrpV1 mRNAs could all be visualized in the mes-di-encephalic area at this stage (Figures 10A1–A4 and Table 2). Calb2, Calb1, and NeuroD6 mRNAs were detected in several brain areas in addition to the mes-di-encephalic area, but TrpV1 mRNA was, apart from rare cells, only identified along the cephalic flexure (Figures 10A1–A4). NeuroD6 mRNA was particularly prominent in the developing cerebral cortex (Figure 10A3) in accordance with a previous publication (Goebbels et al., 2006) and also found in the RMM (Figure 10A3), while Calb2 mRNA was strong in the thalamus (Figure 10A3). Following the same S1-S3 sectioning levels as described in Figure 1, the medial to lateral distribution of each mRNA was analyzed by co-labeling with Th mRNA (Figure 10B). Calb2, Calb1, Neurod6 and Trpv1 all showed individual patterns. Sparse Calb2/Th double-labeling was distributed in the dorsal aspect of the mVTA, in the lVTA and SNc, while prominent Calb2 labeling was also detected outside the Th-positive area, e.g., in the laterally positioned STN (Figures 10B1–B1”; schematic summary displayed to the right for each section level, S1-S3). Calb1/Th double-labeling was seen in the dorsal aspect of the mVTA and some in the lVTA (Figures 10B2–B2”). The NeuroD6 mRNA signal was weak at this stage, and co-labeling with Th was detected in mVTA but not in the lVTA or SNc (Figures 10B3–B3”) in accordance with the medial distribution described in the newborn mouse (Viereckel et al., 2016). Trpv1 mRNA overlapped with Th exclusively in the mVTA, but within the Th-positive area, some Trpv1-positive cells were negative for Th, suggesting a non-dopaminergic phenotype (Figures 10B4–B4”). As expected, the reference mRNA Otx2 was found in Th-positive cells of the mVTA and lVTA but not in SNc (Figures 10B5–B5”), while the reference Girk2 mRNA was sparse and found in both the VTA and SNc at this stage (Figures 10B6–B6”). Foxa1/Th mRNA co-labeling was detected throughout the medial and lateral aspects of VTA with more rare co-labeling in the SNc. In addition, Foxa1 mRNA was prominent in the STN and RN (Figures 10B7–B7”). Finally, Snca mRNA overlapped substantially with Th mRNA within the entire VTA area, but showed less co-localization with Th in the developing SNc at this stage (Figures 10B8–B8”).

Next, co-localization of Calb2, Calb1, NeuroD6, and Trpv1 mRNAs with Th, Vglut2 and Vmat2 mRNAs was analyzed to address putative additional neurotransmitter phenotypes of cells expressing these markers at E14.5. A clear difference in distribution between Calb2 and Calb1 mRNA in the thalamus was observed (Figures 10C1,C2) and the cortical distribution of NeuroD6 mRNA was evident (Figure 10C3). In the midbrain, Calb2, Calb1 and NeuroD6 mRNA overlapped to near-100% with Th mRNA at this stage and also showed occasional overlap with Vglut2 and Viaat mRNAs in the Th-positive area (Figures 10D1–D9 and closeups in Figures 10E1–E9). In contrast, in addition to substantial co-localization with Th mRNA, Trpv1 mRNA co-labeled prominently with both Vglut2 and Viaat mRNAs at E14.5 (Figures 10D10–D12,E1–E12), a pattern similar to previously described at P3 (Viereckel et al., 2016). In summary, at E14.5, TrpV1 mRNA labels a heterogeneous subpopulation of neurons within the medial aspect of the mesencephalon, NeuroD6 mRNA labels a subset of medial and lateral Th-positive and Vglut2-positive neurons and is excluded from the SNc. Calb1 and Calb2 mRNAs show individual patterns in the mVTA and lVTA, and Calb2, but not Calb1, is found in the SNc. Thus, while Grp, Lpl, Fst mRNAs are not detected at either E11.5 or E14.5, mRNAs for NeuroD6, Calb1, Calb2, and TrpV1 label subsets of DA neurons already at E14.5, suggesting that subtype specification has been initiated at this stage.

**FIGURE 11:**
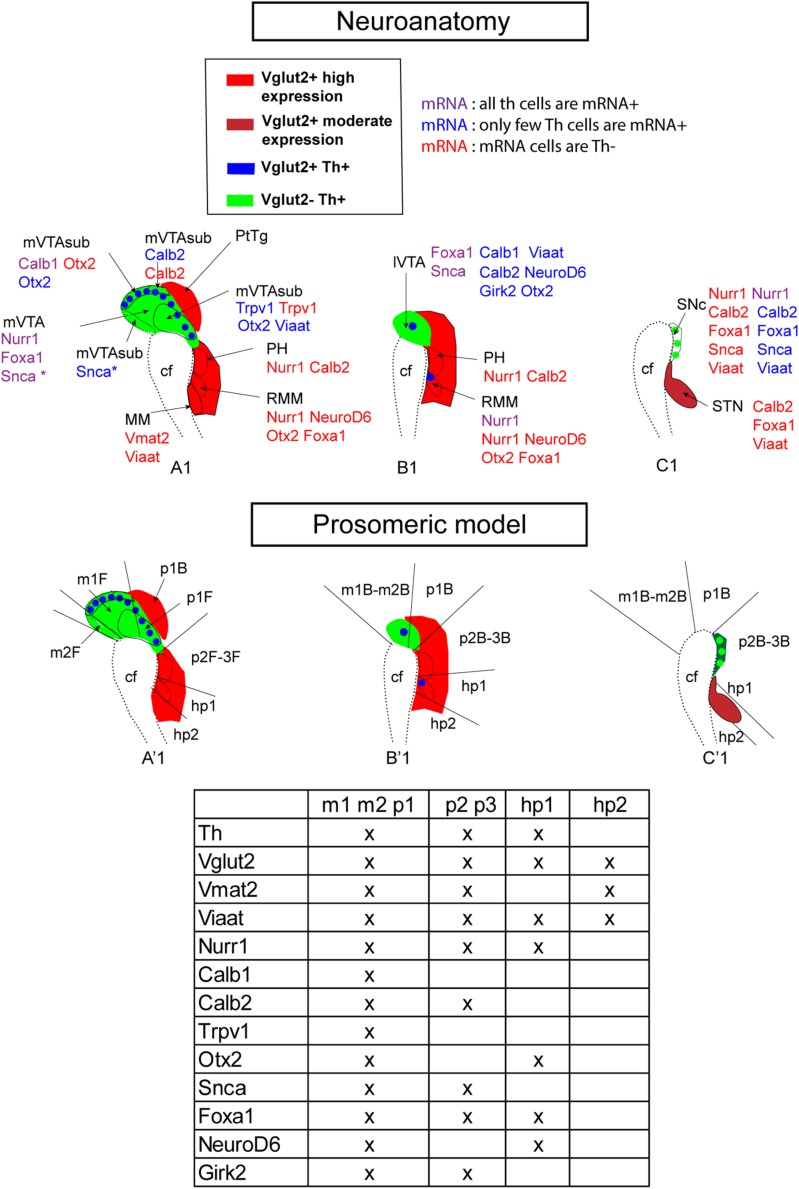
Summary of current mRNA analysis presented in neuroanatomical (top) and prosomeric (bottom) terminology at E14.5. Sagittal sections of the mes-di-encephalic area in the developing mouse embryo at E14.5 illustrating representative section levels S1 **(A1,A’1)**, S2 **(B1,B’1)**, and S3 **(C1,C’1)** as indicated in Figure 1. Legend indicates color scheme for presence of Vglut2 and Vglut2/Th mRNAs. Note Vglut2 mRNA detection of moderate and high level indicated by different shades of red. Additional mRNAs, including vesicular transporter Viaat and Vmat2 (Figures 2, 3), and mRNAs selected for analysis based on presence in dopamine neuron subtypes (Figure 10) shown for each anatomical area (top panel) and prosomer identity (bottom panel, table). In top panel, each mRNA is presented in the context of its degree of co-labeling with Th mRNA (Th mRNA + or Th mRNA-). Caudal to the left; rostral to the right in each picture. cf, cepahlic flexure; hp, hypothalamo-telencephalic prosomer; m, mesomer (mesencephalic prosomer); MM; mammillary nucleus; p, diencephalic prosomer; PH, posterior hypothalamus; R, rostral; RMM, retromammillary nucleus; SNc, substantia nigra *pars compacta*; STN, subthalamic nucleus; VTA, ventral tegmental area; lVTA, lateral VTA; mVTA, medial VTA. (B) Refers to basal plate; F refers to floor plate.

Finally, schematical illustration of developmental brain maps in the context of standard neuroanatomical terminology (Figure 11, top) and the prosomeric model (Figure 11, bottom) allowed a visual mapping of combinations of gene expression patterns at this stage. Schematic illustrations of the histological findings described at E14.5 in mes-di-encephalic brain area, with each mRNA analyzed in the current study compared to Th and how they relate to Vglut2 and Th within the prosomeric model, show how gene expression patterns overlap within differentiating mes-di-encephalic neurons as they adopt a dopaminergic and/or glutamatergic phenotype that together represent distinct subregions or distinct prosomeres.

## Discussion

With the recent realization that mDA neurons exist as multiple subtypes defined by distinct gene expression profiles and that they are located within a strongly heterogeneus surrounding, it is now crucial to pin-point the developmental process leading up to this complexity. By addressing combinations of some newly described and some more established gene expression profiles implementing high spatial resolution analysis at the embryonal stages when DA neurons subtypes are first formed and specified, some unexpected insights into the development of mDA neurons and their neighboring cells were reached: *Firstly*, we identify *Vglut2* expression in the absolute majority of neurons destined to become dopaminergic prior to their expression of *Nurr1* and *Th*, suggesting that *Vglut2* is a marker of early, non-differentiated DA neurons, the function of which remains to be explored. Neither *Viaat* nor *Vmat2* genes show this extensive expression within the developing midbrain, but similar to Vglut2, also Viaat mRNA is found widely distributed throughout the developing brain from early embryogenesis.

*Secondly*, Vglut2 mRNA is to a large extent co-localized with Nurr1 mRNA throughout the mes-di-encephalon, and a dual Vglut2/Nurr1 identity may thus identify both differentiating dopaminergic and glutamatergic neurons within this area. While co-localization is not absolute within the extensive Vglut2/Nurr1-positive continuum, the substantial overlap between Nurr1 and Vglut2 was a surprising finding since the current literature strongly emphasizes *Nurr1* expression in the midbrain as a marker for DA neurons. However, the vast distribution of Vglut2 and Nurr1 mRNAs, forming a partly overlapping continuum stretching from caudal aspect of the mesencepahlon through to the rostral diencephalon is in accordance with single-mRNA labelings reported for Vglut2 and Nurr1, respectively, in the Allen Brain Atlas of the developing mouse embryo^1^. Also previous work focused either on Vglut2 or on Nurr1 in the developing mouse brain has identified the vast mes-di-encephalic distribution of Vglut2 mRNA (Birgner et al., 2010) and Nurr1 mRNA (Smits et al., 2013), respectively. By our double-labeling experiment, we can now directly compare the patterns of Vglut2 and Nurr1 within the same sections, and thereby demonstrate their substantial level of overlap in the mes-di-encephalon. Beyond the mDA system, *Nurr1* expression has previously been reported in several additional brain areas (Zetterström et al., 1996a, b) including the cortex and hippocampus where its expression in glutamatergic neurons was recently implicated in cognitive functions of putative relevance to Alzheimer’s disease (Moon et al., 2019). Taken together, our histological results now suggest that Vglut2/Nurr1 co-localization might be of importance for fate determination of both dopaminergic and glutamatergic neurons within mes-di-encephalic areas.

*Thirdly*, Snca mRNA, encoding the strongly disease-associated protein α-synuclein, co-localizes extensively with Vglut2 and Nurr1 mRNAs in a restricted number of DA neurons already at E10, suggesting a functional role in the early establishment of mDA neurons. Despite the strong implication of aberrant α-synuclein proteins in PD and other synucleinopathies having led to substantial experimental focus on the protein product, the developmental expression pattern of the endogeneous *Snca* gene has remained more elusive. Our identification of Snca mRNA already at E10 should be of interest to explore further, not least in the context of PD and *in vitro* generation of DA neurons.

*Fourthly*, in midbrain DA neurons, NeuroD6 mRNA, but not Grp mRNA, was detected at E14.5, demonstrating that the NeuroD6 and GRP proteins, which have been suggested to partly overlap spatially and to possess pro-survival effects (Chung et al., 2005; Greene et al., 2005; Viereckel et al., 2016; Kramer et al., 2018) have significantly different temporal profiles. The initiation of *NeuroD6* gene expression prior to *Grp* expression may be of importance for any putative attempts aiming to implement their protein products for differentiation and neuroprotection *in vitro*-based DA neuronal systems. Also Calb1, Calb2, Girk2 and TrpV1 mRNAs, representing distinct subpopulations of mes-di-encephalic neurons, showed initiation between E11.5 and E14.5, while several other markers decribed in more mature DA neurons were not yet detectable at these stages. The current findings support recent results of studies implementing scRNAseq analyses of developing DA cells (La Manno et al., 2016; Tiklová et al., 2019) showing that mDA heterogeneity arises early.

### Early Developmental Vglut2/Nurr1 Co-localization in Mes-Di-Encephalic Neurons

In contrast to the intense exploration of mDA development, the developmental programs of midbrain GABA and glutamatergic neurons have been less investigated. Originating from rhombomere 1 rather than the mesencephalic midline, transcription factors were recently identified that confer a balance between inhibitory and excitatory identity in early development (Lahti et al., 2016). Glutamate has been shown to control growth rate of dopaminergic neurons (Schmitz et al., 2009), tentatively suggesting that glutamatergic neurons present within the local brain environment of mDA neurons may be of importance for their development. In the mature brain, VGLUT2-positive neurons of the ventral midbrain send projections to similar target areas as mDA neurons and are involved in similar type of functions (Yamaguchi et al., 2011; Hnasko et al., 2012; Morales and Root, 2014). In addition to neurons that either release DA or glutamate, some DA neurons express the *Vglut2* gene and co-release their DA with glutamate (Trudeau et al., 2014). Temporally, *Vglut2* expression in mDA neurons varies with age and shows stronger expression at birth than at E14.5 (Papathanou et al., 2018). Over-expression of the *Vglut2* gene in PD models has been proposed to serve a neuroprotective role (Dal Bo et al., 2004; Mendez et al., 2008; Steinkellner et al., 2018) while gene-knockout of *Vglut2* in DA neurons significantly alters dopaminergic function, including neuronal survival, DA release, and reward-related behavior (Birgner et al., 2010; Hnasko et al., 2010; Stuber et al., 2010; Alsiö et al., 2011; Fortin et al., 2012; Wang et al., 2017; Papathanou et al., 2018).

The behavioral phenotype is weaker when *Vglut2* is deleted from DA neurons in adulthood than during embryogenesis, further emphasizing a developmental role for *Vglut2* in DA neurons (Papathanou et al., 2018). A full knock-out of *Vglut2* gene expression, which affects both glutamatergic neurons and glutamate co-releasing neurons, causes post-natal death due to respiratory deficiency, however, gross anatomical analysis did not reveal any major discrepancies in the mDA system (Wallén-Mackenzie et al., 2006).

While the temporal onset and spatial distribution of endogenous *Vglut2* gene expression at early developmental stages has remained largely unexplored, our histological results, using Tph/Vmat2 (RN) as caudal border and Pitx2 (STN and pSTN) as rostral border, now show that even prior to *Th*-expression, *Vglut2* is prominently expressed in a medial continuum stretching from the caudal aspect of the ventral mesencephalon into the rostral part of the ventral diencephalon, covering an area that develops into multiple cell groups of dopaminergic and glutamatergic phenotype. Based on analysis starting from E9.5, our histological gene expression analysis suggests that Vglut2-positive neurons develop into Nurr1-positive neurons which in turn give rise to both Nurr/Th double-positive DA neurons and Th-negative Vglut2/Nurr1 double-positive glutamatergic neurons. Vglut2-positive Th neurons may thus represent a developmentally primitive form of DA neurons: While all, or at least the majority, of Th-positive neurons in the ventral midbrain express the *Vglut2* gene when the *Th* gene expression is first turned on, *Vglut2* is progressively down-regulated upon *Th* expression within the majority of DA neurons. Histological analyses provide a snap-shot of the time-point when animals are sacrificed, however, by systematic analysis of multiple embryos covering a short, but critical embryonal time-period, present data propose that the *Vglut2* gene is expressed in most early differentiating DA neurons (E9.5-E11) to be downregulated around E11.5-E14.5. Already at E11.5 and E14.5, we find less co-localization between Vglut2 and Th mRNAs than at E10-11.

Thus, while *Vglut2* expression in maturing DA neurons is highly modest, pre-dopaminergic and early dopaminergic neurons, as well as developing non-dopaminergic neurons within the area, show substantial mRNA encoding this transporter. We tentatively hypothesize that Th-positive neurons gradually lose their Vglut2 phenotype when they start to migrate, first ventrally and then tangentieally toward their final position in the mVTA, lVTA and the lateral-most located SNc, which is devoid of Vglut2. Observations that support this idea include: There is an increase in the mDA surface at the medial level from E10.5 to E14.5 which seems to be linked with the increase of the mantle surface; the main mVTA subregion where Th/Vglut2 co-localization are detected is the caudo-dorsal aspect of the mVTA, next to the ventricle in which Th neurons are born; the thickness of the mantle increase as Th neurons start to migrate dorso-ventrally and subsequently, tangetially. Together with reports of its plasticity and putative pro-survival properties (Dal Bo et al., 2004; Mendez et al., 2008; Fortin et al., 2012; Steinkellner et al., 2018), the present results suggest that *Vglut2* expression may be of importance both for initial specification of mDA neurons and for their maintenance during challenge, roles that should be explored further.

Current knowledge of the developmental programs for appropriate differentiation of the dopaminergic phenotype has advanced regenerative medicine, and today, several protocols exist for *in vitro* generation of DA neurons from various sources of stem/progenitor cells, or by reprograming of somatic cells (Henchcliffe and Parmar, 2018). The finding that *Nurr1* is broadly expressed in both dopaminergic and glutamatergic neurons throughout the mes- and di-encephalon *in vivo* might be of importance when using Nurr1 as verification marker for the dopaminergic phenotype. Within this same context, a glutamatergic phenotype (defined by *Vglut2* expression) has been considered a contamination when generating DA neurons (Kee et al., 2017; Kirkeby et al., 2017). Indeed, when utilizing *in vitro* protocols aimed at generating DA neurons, obtaining a glutamatergic phenotype might lower the DA ratio, and it could therefore be critical to deselect a glutamatergic phenotype early in the differentiating process. However, deselecting neurons positive for glutamatergic markers such as Vglut2 may directly counteract attempts to mimic the appropriate environment for differentiation. The extensive overlap between Vglut2 and Nurr1 mRNAs in neurons destined to become either dopaminergic or glutamatergic might thus important to consider when implementing expression of these genes as selection markers.

### The NeuroD6 VTA DA Neuron Subtype, Recently Implicated in Neuroprotection and Behavioral Reinforcement, Appears Already at E14.5 Along Some Other Subtypes

By analysis at E11.5 and E14.5, our study follows the same timeline as implemented in a recent scRNAseq study of mice, human and stem cells, where these developmental stages in mice, corresponding to weeks 6 and 10 in the human, showed similar gene expression results between DA cells obtained from brains of mice and humans (La Manno et al., 2016). In the study by La Manno et al. (2016), three subsets of neurons were identified at these stages: (DA0) defined by proneural genes and *Th*; (DA1) additional genes including the *Dopamine transporter* (*Dat*); (DA2) other marker genes. Placing our current histological data of v-transporters within the midbrain in the context of these time-points, we find that Viaat, Vglut2, and Vmat2 mRNAs can all be detected at both E11.5 and E14.5. Notably, Viaat, but not Gad1 and Gad2, mRNAs were detected at E11.5 while all three were found at E14.5. Viaat was almost entirely excluded from mDA neurons, whereas Vglut2 mRNA, as discussed above, forms a prominent developmental mes-di-encephalic continuum stretching caudally from the RN into diencephalic structures. The Vglut2-positive area includes the entire mesencephalic DA area at E11.5, but primarily the mVTA at E14.5. At E11.5, Th/Vglut2 co-labeling is substantially higher than Th/Vmat2 as Vmat2 mRNA is still low in the midbrain at this early stage. In contrast, at E14.5, Th/Vmat2 show substantial overlap in the midbrain, while at this stage, only a modest number of Th-positive neurons remain positive for Vglut2 mRNA.

Further, scRNAseq analysis has shown that five subtypes of mDA neurons can be identified by combinatorial gene expression in the mature brain, including one for the SNc subtype and four for VTA (VTA1-4) (Poulin et al., 2014; La Manno et al., 2016). Notably, within VTA(2), the genes encoding NeuroD6 and GRP were enriched. In the present study, we identified NeuroD6, but not Grp mRNA, at E14.5 while neither was detected at E11.5. NeuroD6 and GRP have been reported as potential neuroprotective factors that may contribute to the enhanced survival of certain VTA neurons in PD and experimental models of this disorder (Chung et al., 2005; Greene et al., 2005; Kramer et al., 2018). NeuroD6 KO mice have reduced number of midbrain DA neurons, showing that this basic-loop-helix type transcription factor is crucial for normal DA cell development (Khan et al., 2017). Beyond roles in differentiation and survival, we could recently show that activation of NeuroD6-positive DA neurons induces behavioral reinforcement, an important aspect of motivated behavior (Bimpisidis et al., 2019). By pinpointing that distinct features of reward-related behavior can be regulated by NeuroD6 subtype DA neurons, dysfunction of this subtype may be directly correlated with certain symptoms of neuropsychiatric disorder. Developmental correlation is critical in the context of neuropsychiatric and neurological conditions, and with detailed developmental mapping, new knowledge of developmental specifications, including molecular markers, might help in advancing diagnosis and prevention.

The analyses performed in this study have been presented using standard nomenclature of many brain atlases and publications. However, to align with current terminology of the prosomeric model, formulated on the basis molecular and experimental evidence (Puelles and Rubenstein, 2003, 2015; Marín et al., 2005; Puelles, 2019), our results have been addressed also in the context of this nomenclature. The prosomeric model is an updated version of the neuromeric model, based on neuromeres, which are longitudinally organized segments that are additionally regionalized in the dorso-ventral axis (reviewed in Puelles, 2019). By addressing our *in situ* hybridization results in the context of prosomeres, mRNA patterns within distinct prosomeres could be identified. Contrary to the columnar model, the prosomeric model has placed the VTA and SNc in both the mes- and diencephalon, and this has been taken into account in the presentation of the data. By analyzing our results using both standard neuroanatomy and prosomeric terminology, we also provide examples of developmental brain maps, created to show how gene expression patterns overlap within differentiating mes-di-encephalic neurons as they adopt a dopaminergic and/or glutamatergic phenotype (Figures 9, 11). For example, the combination Calb2/Vglut2 separates p2,3 from hp1 which is positive for NeuroD6/Vglut2 and negative for Calb2. Another example, Vglut2/Nurr1 overlap was strong in the m1, m2, p1, p2, p3, and hp1, encompassing mDA and several diencephalic glutamatergic neuronal population, while hp2 (including the MM) was devoid of Nurr1, but instead positive for Vglut2/Vmat2 co-labeling. Indeed, the analysis of multiple molecular markers using many combinations of double-fluorescent *in situ* hybridization enabled the identification of several subregions that can be identified in the developing mVTA that distinguish them from each other, and from the lVTA and SNc already at early stages.

Genes expressed within developing DA neurons have been carefully analyzed to unravel the developmental program for their generation, specification and survival. However, the natural brain environment, in which DA neurons do not exist in isolation but are part of a heterogeneous brain environment, is rarely taken into consideration when studying the developmental programs of DA neurons. By implementing systematic histological analysis of the brain environment in which DA neurons develop, the current study offers some insight into the natural brain area of developing DA neurons. Our results lead us to suggest that the mes-di-encephalic area gives rise to Vglut2-positive neurons that subsequently express the *Nurr1* gene and that these Vglut2/Nurr1-double positive neurons in turn give rise both to dopaminergic and glutamatergic neurons of different subtypes. While our results are based on mRNA localization within brain tissue, mechanistic approaches will be required to pin-point any regulatory relationships.

## Data Availability Statement

All datasets generated for this study are included in the article/Supplementary Material.

## Ethics Statement

The animal study was reviewed and approved by the Ministère de l’Agriculture et de la Forêt, Service Vétérinaire de la Santé et de la Protection Animale (Permit No. A 94-028-21).

## Author Contributions

SD performed all the experiments, analyzed the data, prepared the figures, and reviewed the text. ÅW-M conceived the study and was in charge of overall planning and design, analyzed the data, prepared the figures, and wrote the text.

## Conflict of Interest

SD is the owner of Oramacell, Paris. The remaining author declares that the research was conducted in the absence of any commercial or financial relationships that could be construed as a potential conflict of interest.

## Footnotes

1 http://developingmouse.brain-map.org

## Supplementary Material

FIGURE S1 Medial-lateral distribution of Nurr1, Th, Vglut2 mRNA in ventral mes-di-encephalon at E14.5. Serial sagittal sections from medial to lateral aspect of brain showing overview of brain. mRNA for Nurr1 (red)/Th (green) **(A1–A5)**; Nurr1 (red)/Vglut2 (green) **(B1–B5)**; Vglut2 (red)/Th (green) **(C1–C5)** followed. Scale bars: 500 μm. C, caudal; CTM, continuum; R, rostral; SNc, substantia nigra *pars compacta*; VTA, ventral tegmental area; lVTA, lateral VTA; and mVTA, medial VTA.
