# Supplementary figures and images for: Developmental Co-expression of Vglut2 and Nurr1 in a Mes-Di-Encephalic Continuum Preceeds Dopamine and Glutamate Neuron Specification
Source: Front Cell Dev Biol. 2019 Nov 28;7:307. doi: 10.3389/fcell.2019.00307 (PMC6892754; doi:10.3389/fcell.2019.00307)

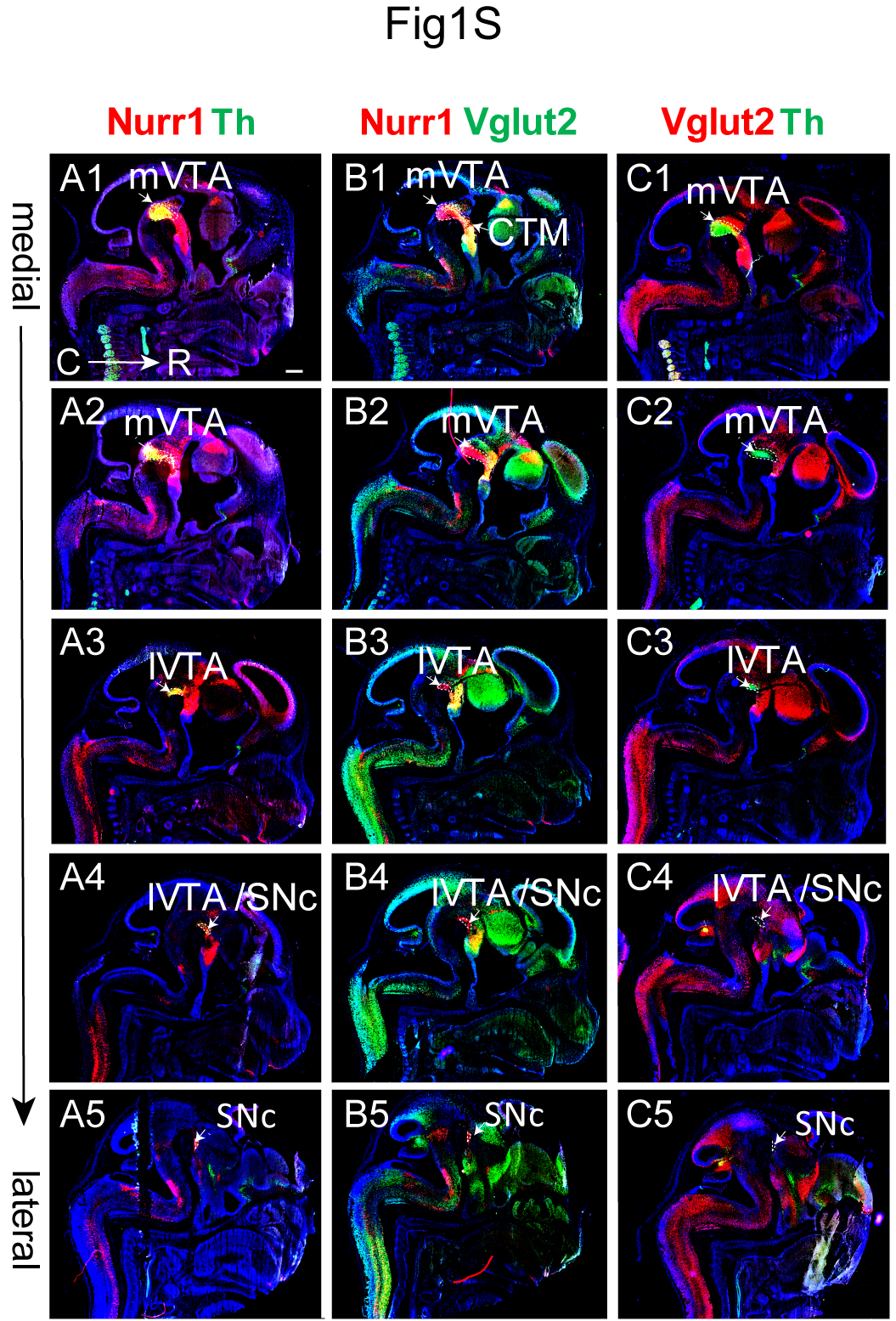

Supplement: FIGURE S1 — Medial-lateral distribution of Nurr1, Th, Vglut2 mRNA in ventral mes-di-encephalon at E14.5. Serial sagittal sections from medial to lateral aspect of brain showing overview of brain. mRNA for Nurr1 (red)/Th (green) (A1–A5); Nurr1 (red)/Vglut2 (green) (B1–B5); Vglut2 (red)/Th (green) (C1–C5) followed. Scale bars: 500 μm. C, caudal; CTM, continuum; R, rostral; SNc, substantia nigra pars compacta; VTA, ventral tegmental area; lVTA, lateral VTA; and mVTA, medial VTA. [file Image_1.tif]
